# *PTM‐Psi* : A python package to facilitate the computational investigation of *p* ost‐ *t* ranslational *m* odification on *p* rotein *s* tructures and their *i* mpacts on dynamics and functions

**DOI:** 10.1002/pro.4822

**Published:** 2023-12-01

**Authors:** Daniel Mejia‐Rodriguez, Hoshin Kim, Natalie Sadler, Xiaolu Li, Pavlo Bohutskyi, Marat Valiev, Wei‐Jun Qian, Margaret S. Cheung

**Affiliations:** ^1^ Physical Sciences Division, Physical and Computational Sciences Directorate, Pacific Northwest National Laboratory Richland Washington USA; ^2^ Biological Sciences Division, Earth and Biological Sciences Directorate, Pacific Northwest National Laboratory Richland Washington USA; ^3^ Biological Systems Engineering Washington State University Richland Washington USA; ^4^ Environmental Molecular Sciences Laboratory Richland Washington USA; ^5^ University of Washington Seattle Washington USA

**Keywords:** molecular docking, molecular dynamics, quantum mechanics, redox proteome

## Abstract

Post‐translational modification (PTM) of a protein occurs after it has been synthesized from its genetic template, and involves chemical modifications of the protein's specific amino acid residues. Despite of the central role played by PTM in regulating molecular interactions, particularly those driven by reversible redox reactions, it remains challenging to interpret PTMs in terms of protein dynamics and function because there are numerous combinatorially enormous means for modifying amino acids in response to changes in the protein environment. In this study, we provide a workflow that allows users to interpret how perturbations caused by PTMs affect a protein's properties, dynamics, and interactions with its binding partners based on inferred or experimentally determined protein structure. This Python‐based workflow, called *PTM‐Psi*, integrates several established open‐source software packages, thereby enabling the user to infer protein structure from sequence, develop force fields for non‐standard amino acids using quantum mechanics, calculate free energy perturbations through molecular dynamics simulations, and score the bound complexes via docking algorithms. Using the *S*‐nitrosylation of several cysteines on the GAP2 protein as an example, we demonstrated the utility of *PTM‐Psi* for interpreting sequence–structure–function relationships derived from thiol redox proteomics data. We demonstrate that the *S*‐nitrosylated cysteine that is exposed to the solvent indirectly affects the catalytic reaction of another buried cysteine over a distance in GAP2 protein through the movement of the two ligands. Our workflow tracks the PTMs on residues that are responsive to changes in the redox environment and lays the foundation for the automation of molecular and systems biology modeling.

## 1 INTRODUCTION

Post‐translational modification (PTM) is a critical mechanism for regulating protein functions by chemically modifying the side chains of amino acids (Paulsen & Carroll, 2013). These site‐specific PTMs on the protein structures perturb or even modify the activity state (Zhang et al., 2021), the Energy Landscape (Garrido Ruiz et al., 2022), and the dynamics that affect functions by regulating the signaling processes in profound ways (Harris et al., 2020; Liu et al., 2022; Qin et al., 2020). While some PTMs, such as phosphorylation, require specific enzymes to characterize cellular behaviors, oxidation–reduction (redox)‐based PTMs (i.e., redox PTMs) (Kitatani et al., 2006) of thiol‐containing cysteines (Jumper et al., 2021) are the most common in microbes for regulating metabolic pathways for their rapid responses to shifting redox conditions. These cysteine‐mediated redox PTMs target and modify the function, localization, and/or activity of widespread proteins in a strictly regulated and often reversible manner (Paulsen & Carroll, 2013), depending on their positions in a protein. It remains challenging to gain spatial information about the positions of PTMs on cysteines without elaborate experimental designs (Zhang et al., 2021) due to a wide variety of possible combinations of modifying redox PTMs on thiol‐containing cysteines.

Mass spectrometry (MS)‐based redox proteomics has enabled quantitative profiling of redox proteomics for the measurement of cysteine thiol PTMs stoichiometrically (Ahdritz et al., 2022). However, because multiple PTM forms, potentially transient or labile (Qin et al., 2020), can be present at a single cysteine site, the coverage of cysteine oxidation in protein structures, biophysics, and simulations is still an underdeveloped area (Garrido Ruiz et al., 2022). One of the major hurdles in computer simulations is the highly electronegative cysteine thiols that react swiftly with the reactive oxygen species in the local environment. Recently, the reactiveness of the protonated states of cysteines has been investigated by using constant pH molecular dynamics (MD) in the implicit solvent model (Harris et al., 2020) and used for the design of small molecules or inhibitors (Liu et al., 2022). It is challenging to evaluate thiol PTMs on protein structures and properties using MD simulations because the diverse oxidated PTMed forms are not part of the standard force fields. Occasionally, when experimentally determined protein structures with distinct PTMs (Garrido Ruiz et al., 2022) were available, comparative investigations performed using MD simulations demonstrated the impact of PTMs on protein dynamics. Such an effect can potentially alter the molecular interactions between proteins and promote or impede the formation of quaternary complexes. Other quantum mechanical (QM) approaches were also used for modeling PTMs on enzymatic reactions (Qin et al., 2020). However, the scope of these computer simulations is often limited to the structural arrangement of the surrounding atoms at the reaction site.

To streamline the quantitative analysis of PTMs on protein dynamics, structures, and interactions, we have developed a computational workflow—*PTM‐Psi*—to fulfill the need of non‐standard force fields for PTMed cysteines from QM calculations as we bridged the technical gap between protein structure inference and the MD simulations. *PTM‐Psi* is offered in a user‐friendly Python package that helps researchers interpret the functional annotation of diverse PTMs, not limited to thiol PTMs, on protein structures. As a use case study, we focused on thiol PTM on a metabolic enzyme because its modeling requires chemical intuition and manual trial and error, and the accurate simulation of the modeled system will become intractable when the reactions are considered in a proteome level, including all possible thiol modifications.

**FIGURE 1:**
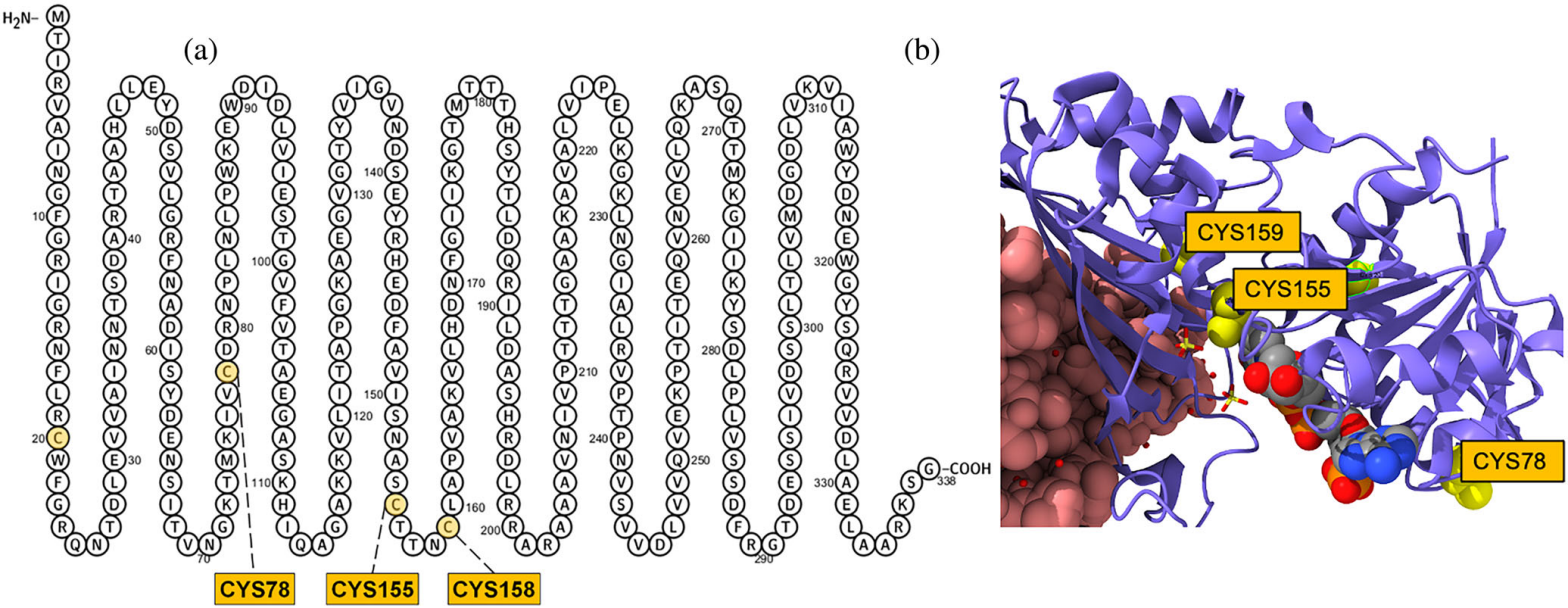
(a) The amino acid sequence of the GAP2 monomer with the locations of three cysteines (CYS 78, 154, and 159, highlighted in yellow circles) chosen to be *S*‐nitrosylated. (B) The structural representation of a single GAP2 subunit with the three labeled cysteines and its ligand.

The metabolic enzyme we took as a use case example to demonstrate the utility of *PTM‐Psi* is nicotinamide adenine dinucleotide phosphate (NADP)‐dependent glyceraldehyde‐3‐phosphate dehydrogenase (also known as homotetramer NADP‐GADPH protein from *Gap2* gene; Kitatani et al., 2006) of *Synechococcus elongatus PCC 7942* as an example (Figure 1), referred as “GAP2”). The utility of *PTM‐Psi* starts with a structure that could either be inferred by AlphaFold2 or be an experimentally determined one from the protein databank (PDB). The following steps involve the parameterization of non‐standard force fields for nitrosylated cysteines from electronic structures calculated via NWChem. Next, the workflow is then followed by the analysis of free energy perturbation to evaluate the impact of nitrosylated thiols on protein dynamics using a thermodynamic integration approach with all‐atom classical MD simulations performed by GROMACS simulation package. The workflow has a module allowing the users to rank the impact of nitrosylated thiols on the protein–ligand interactions using the molecular docking software Autodock to further the interpretability of the data from the structural dynamics. Here, by elucidating how *S*‐nitrosylated cysteines affect GAP2's folding stability and ligand binding using *PTM‐Psi*, we showed its utility of providing probable hypotheses for experimentalists to further interpret the relationship of protein's sequence, structure, and function on the redox proteomic datasets from MS.

## 2 RESULTS

### 2.1 PTM‐Psi workflow

**FIGURE 2:**
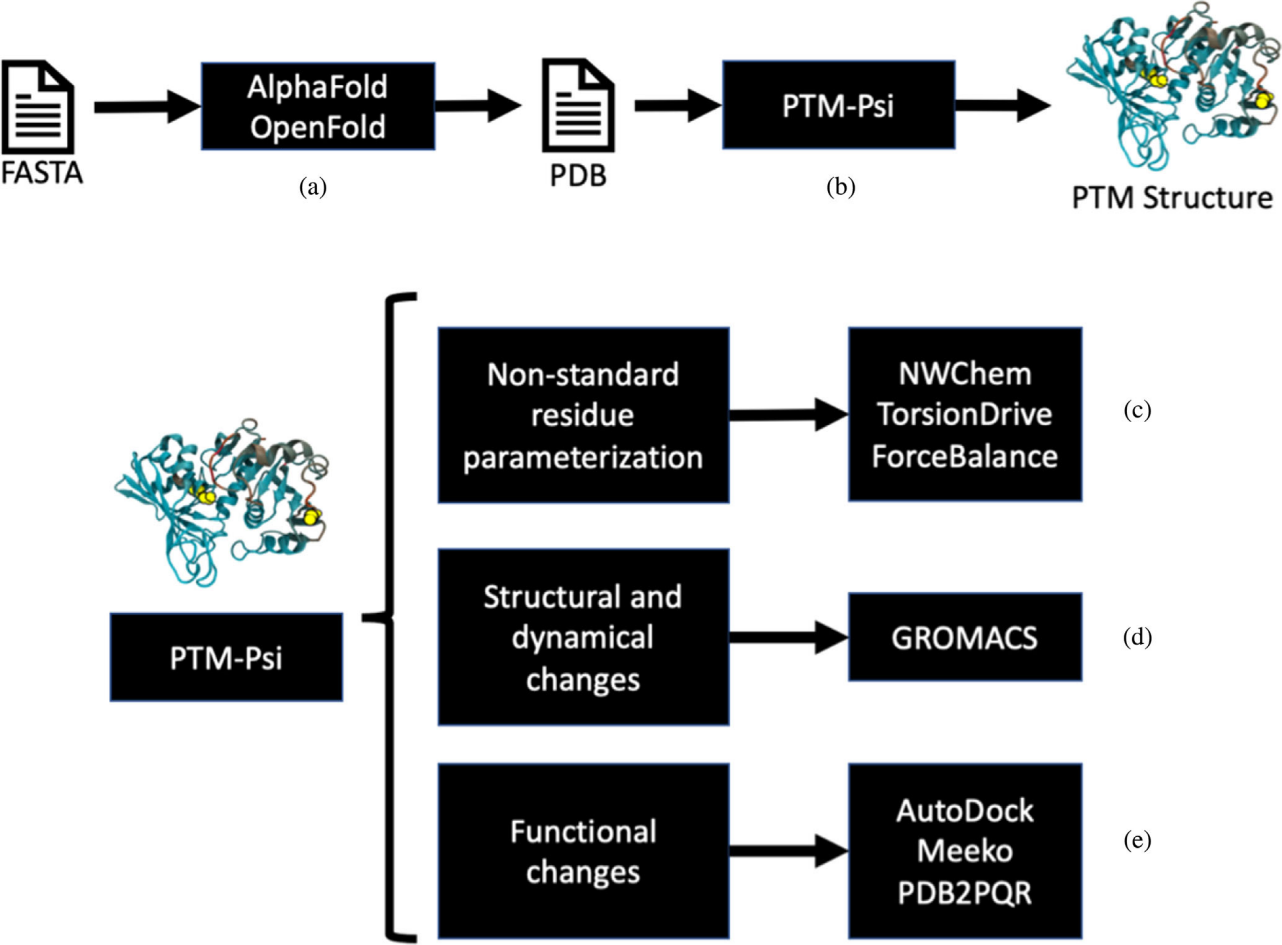
The workflow of the *PTM‐Psi* software package includes input files and launch instances from standard packages such as AlphaFold, NWChem, GROMACS, and the Autodock Suite. The workflow can start from (a) a FASTA sequence to perform structural inference using OpenFold or AlphaFold or from a PDB file. (b) *PTM‐Psi* assigns a targeted PTM to the structure. (c) *PTM‐Psi* produces necessary input files to parameterize non‐standard residues using NWChem as the quantum‐mechanics backend. (d) Once all force field parameters are at hand, *PTM‐Psi* prepares GROMACS molecular dynamics and provides standard protocols to evaluate the impact of PTM on the thermodynamics and the structural changes of proteins. (e) *PTM‐Psi* prepares input files for small‐molecule docking simulations using the Autodock Suite to interrogate functional changes, such as the structural compatibility of a substrate to fit into a catalytic site. *PTM‐Psi*, *p* ost‐ *t* ranslational *m* odification on *p* rotein *s* tructures and their *i* mpacts.

*PTM‐Psi* combines several capabilities to streamline the workflow to interrogate the impact of PTMs on proteins using well‐established software packages (Figure 2).

The *PTM‐Psi* workflow starts from single or multiple FASTA sequences for structural inference, using either AlphaFold (Jumper et al., 2021), OpenFold (Ahdritz et al., 2022), or from a PDB file in the local filesystem, the PDB website (https://rcsb.org), or the AlphaFold Structural Database (https://alphafold.ebi.ac.uk/), as indicated in Figure 2a. *PTM‐Psi* can introduce several PTMs into a protein (Figure 2b **)** and prepare topology and force field input files for running MD simulations. If no standard force field parameters are available, *PTM‐Psi* prepares the input files necessary to obtain the missing parameters using NWChem (Apra et al., 2020) as the QM backend, as shown in Figure 2c. Once the force fields for standard or non‐standard amino acids are generated, the workflow examines the impact of PTMs using free energy perturbation calculations based on the GROMACS MD simulation package (Abraham et al., 2015) (Figure 2d **)**. The workflow also assesses the impact of PTMs on protein–ligand binding affinity using either Autodock (Apra et al., 2020) or Autodock‐GPU (Santos‐Martins et al., 2021), as shown in Figure 2e (Apra et al., 2020).

### 2.2 Software details

The *PTM‐Psi* package can be installed using the source code available in the GitHub repository: https://github.com/pnnl/ptmpsi. In the near future, we plan to make *PTM‐Psi* available through other common channels, like the Python Package Index, conda‐forge, and Singularity containers. The GitHub repository contains simple instructions on how to clone and install *PTM‐Psi* inside a virtual environment using the **PIP** Python package manager.

*PTM‐Psi* will produce SLURM (Yoo et al., 2003) batch scripts with all necessary commands to obtain a given outcome. The SLURM scripts produced by *PTM‐Psi* default to match the environment of the Tahoma high‐performance computing (HPC) cluster of the Environmental Molecular Science Laboratory. These scripts are fully customizable by the user using keyword arguments in order to adapt them to another HPC environment. This design choice allows the user to inspect the created input files and submission scripts, add non‐customizable options, and correct any errors in the generated files.

It is important to mention that *PTM‐Psi* does not perform costly computations by itself. Instead, it includes basic functions to manipulate structures and generate complex workflows, calling other more specialized software packages. Sensible default parameters will allow the potential user to take a hands‐off approach, saving human time and reducing the time invested to learn the particularities of many different codes.

#### 2.2.1 Manipulation of protein structures

An unknown three‐dimensional protein structure can be inferred from protein sequences using either AlphaFold or OpenFold. The inference method of the alphafold and openfold submodules accepts a list of FASTA sequences either as a list of strings or as a list of paths. The inference method will produce a SLURM script with the following workflow: (1) creates a Python virtual environment, (2) installs Python dependencies, (3) pulls an appropriate singularity container from the GitHub container registry, (4) runs inference, and (5) copies results back to submission directory. The only requirement to run AlphaFold or OpenFold is to have the database files available. No local installation of the actual programs is needed as they are pulled as Singularity packages. The default behavior of the inference method is to use the monomer model. Consequently, AlphaFold or OpenFold will produce as many monomer structures as the number of FASTA sequences passed to the method. The user can override this behavior by means of the model keyword argument.

If a three‐dimensional structure is already known, the user can rely on the Protein class to download the structure from the PDB or the AlphaFold Structural Database or to read a local PDB file. For example, the snippet



gap2_1 = ptmpsi.protein.Protein(pdbid="2d2i")



gap2_2 = ptmpsi.protein.Protein(uniprotid="q9r6w2")



gap2_3 = ptmpsi.protein.Protein(pdb="6gfp.pdb")




will load three different models for the GADPH enzyme of cyanobacteria. The first line will fetch the model from the PDB, the second from the AlphaFold Structural Database, and the third one will try to read a local file. Loaded structures can be extended using the prepend and append methods of the Protein class. Cartesian coordinates of the new residues are generated using a combination of the Natural Extension of Reference Frame (NERF) algorithm (Parsons et al., 2005) and a quaternion‐based coordinate alignment algorithm. If no *φ* or ψ torsion is specified, *PTM‐Psi* will default to the extended β‐strand conformation. Before adding the new atoms, *PTM‐Psi* will check for and try to avoid clashes with pre‐existing ones. In order to do so, the sidechain *χ*_1_ and *χ*_2_ torsion angles are scanned by *PTM‐Psi* in 30° increments. The scanning process stops at the first *χ*_1_/*χ*_2_ combination for which no clashes are detected. If clashes cannot be avoided, *PTM‐Psi* will still produce a modified structure using the conformation with the least number of clashes. Should this happen, it will be the user's responsibility to assess the validity of such a structure. The prepend and append methods are useful to cap the *N*‐terminus with an acetyl group (ACE) or the C‐terminus with an *N*‐methyl group (NME).

Missing hydrogen atoms can be added using the protonate method of the Protein class. Protonation states are assigned using the propKA method (Olsson et al., 2011; Sondergaard et al., 2011) implemented in the PDB2PQR Python package (Dolinsky et al., 2004, 2007).

The mutate method of the Protein class can be used to introduce point mutations in the protein structure. *PTM‐Psi* keeps a library of standard and non‐standard amino acids that can be chosen to replace an existing residue. The mutate method first aligns the backbones, then rotates the *χ*_1_ dihedral in order to align the β‐carbon atoms. If clashes are detected, *PTM‐Psi* will perform a rotamer search by rotating the *χ*_1_/*χ*_2_ dihedral angles and will select the structure with the lowest number of clashes. Two input arguments are required by the mutate method: a string identifying the residue to be mutated and a string with the three‐letter code of the residue replacing it, as shown in the following example.



gap_2.mutate("A:CYS155", "MET")






The string that identifies the residue to be mutated is composed of the name of the chain, a semicolon, the three‐letter residue name, and the residue number in the chain sequence. It is also possible to identify the mutation site by omitting the three‐letter residue name, i.e., using a string like “A:CYS155” in the example above.

#### 2.2.2 PTM assignment

*PTM‐Psi* introduces post‐translation modifications by using either the mutate or modify methods of the Protein class. *PTM‐Psi* contains a library of common Cys PTMs, saved as non‐standard amino acids, that can be used with the mutate method: *S*‐nitroso‐l‐cysteine (SNC), *S*‐mercapto‐l‐cysteine (CSS), *S*‐methyl‐l‐cysteine (SMC), *S*‐cyano‐l‐cysteine (XCN), *S*‐hydroxy‐l‐cysteine (CSO), l‐cysteine sulfinate (CSD), l‐cysteine sulfonate (OCS), *S*‐carbamoyl‐l‐cysteine (QCS), *S*‐glutathionyl‐l‐cysteine (CGL), and l‐cystine (IYY). These three‐letter codes were obtained from the Chemical Component Dictionary (Westbrook et al., 2015).

The modify method of the Protein class takes a slightly different approach. *PTM‐Psi* uses a library of small radicals that can be attached to some of the standard amino acid residues. The NERF algorithm is used to generate the Cartesian coordinates of the newly introduced moiety. Available modifications include the attachment of methyl, acetyl, and phosphonate (sometimes referred to as phosphoryl) radicals for many amino acids, as well as all radicals to generate the same modified CYS residues available with the mutate command. The syntax of the mutate and modify methods is similar; however, the modify method expects the name of the chemical reaction in the second argument. For example,



gap_2.modify("A:SER154", "acetylation")




acetylates the hydroxy group of SER154. Table 1 shows all valid combinations currently accessible by the modify method. Amino‐terminal acetylation of any residue can be accessed through the prepend command and, to avoid confusion with other side‐chain acetylations is not accessible using the modify command and is not listed in Table 1.

**TABLE 1:** List of post‐translation modifications currently accessible through the modify command of the *PTM‐Psi* package.

Amino acid	Modification
ARG	Methylation
	Symmetric demethylation
	Asymmetric demethylation
	Phosphorylation
ASP	Phosphorylation
CYS	Carbamylation (carbamoylation)
	Cyanylation
	Cysteinylation
	Glutathionylation
	Methylation
	Nitrosylation (nitrosation)
	Phosphorylation
	Sulfenylation
	Sulfhydration
	Sulfinylation
	Sulfonylation
GLU	Methylation
HIS	Methylation
	Phosphorylation
LYS	Acetylation
	Methylation
	Dimethylation
	Trimethylation
	Phosphorylation
SER	Phosphorylation
THR	Phosphorylation
TYR	Phosphorylation

Abbreviations: ARG, arginine; ASP, aspartic acid; CYS, cysteine; GLU, glutamic acid; HIS, histidine; LYS, lysine; *PTM‐Psi*, *p*ost‐*t*ranslational *m*odification on *p*rotein *s*tructures and their *i*mpacts; SER, serine; THR, threonine; TYR, tyrosine.

**FIGURE 3:**
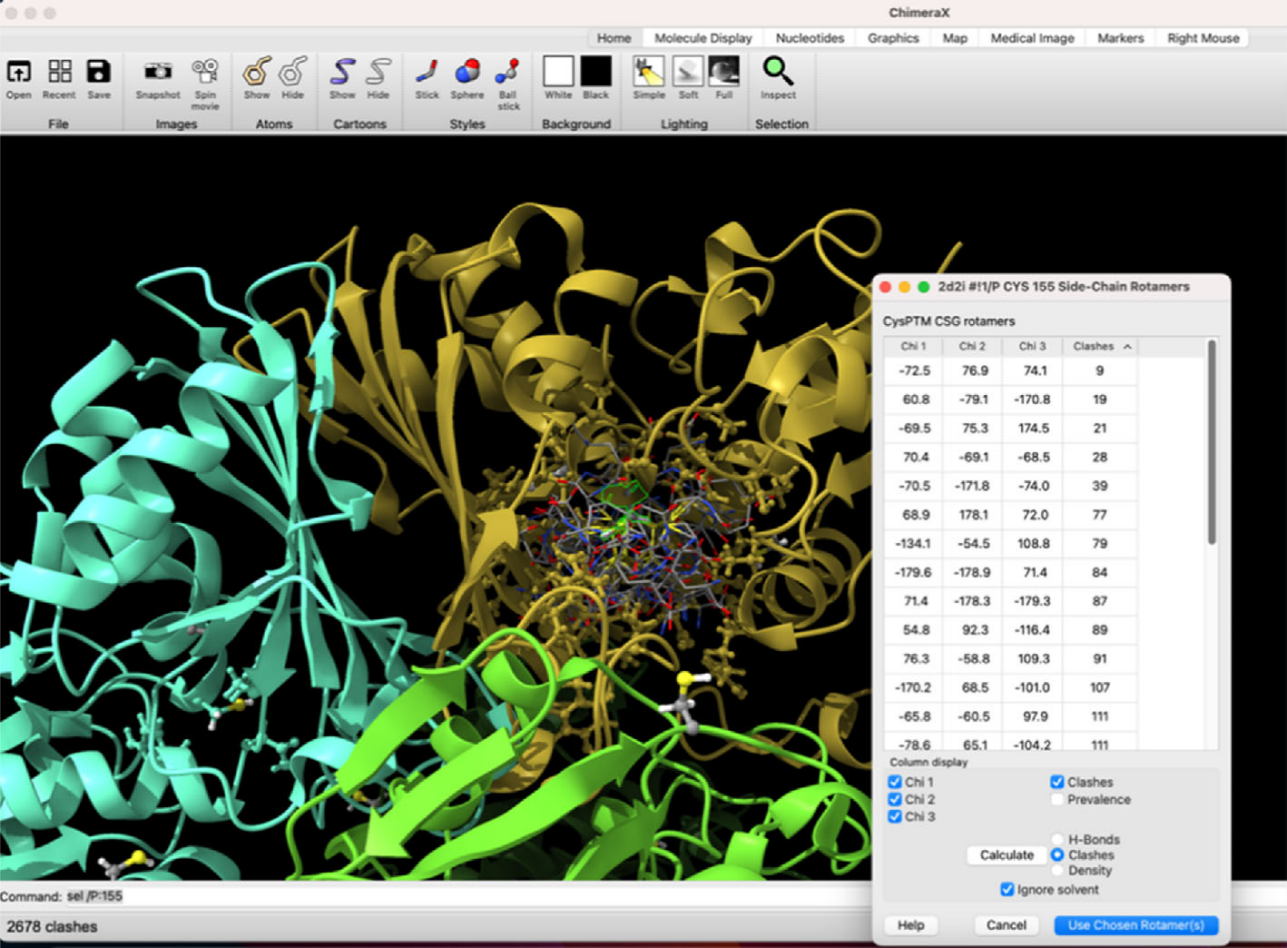
The *doPTM* and *CysPTM* ChimeraX plugins enable the introduction of specific cysteine post‐translation modification in an interactive manner. The picture shows the glutathionylation of the CYS155 (active site) residue of the GAPDH (“GAP2”) protein from *Synechococcus elongatus*.

The *PTM‐Psi* GitHub repository also contains two plugins for the ChimeraX molecular visualization package (Pettersen et al., 2021) for the interactive introduction of cysteine PTMs (Figure 3). The plugins enable a new tool called *doPTM* with functionality very similar to the existing *Rotamer* tool. However, *doPTM* swaps a cysteine for one of the common post‐translationally modified residues listed in the Supporting Information. We plan to extend the *doPTM* tool's capability to support a wider array of PTMs. One of the advantages of using the *doPTM* plugin in ChimeraX is that the most suitable rotamer can be selected based not only on the number of clashes but also based on the number of hydrogen bonds in which the rotamer might be involved. Another advantage is the use of rotamer libraries to avoid the need for dihedral scans, which can be time‐consuming. Detailed information about all of these steps is provided in the Method and Supplementary Information.

#### 2.2.3 Generation of force field parameters

The *PTM‐Psi* package can generate restrained electrostatic surface potential (RESP) charges (Bayly et al., 1993; Cieplak et al., 1995; Cornell et al., 1993) and bonded parameters compatible with the Amber FF99SB (Hornak et al., 2006) and FF14SB (Maier et al., 2015) forcefield families. NWChem is used as a backend to obtain the reference ab initio data. We chose to split the force field parameterization into two steps to allow finer control by the user: (1) generation of QM data and calculation of RESP charges, bond, and angle force constants and (2) fitting of the torsion potential parameters.

The get_qm_data method of the nwchem submodule accepts a residue instance and generates the α and β conformers of the corresponding *N*‐acetyl‐XXX‐*N*′‐methylamine (ACE‐XXX‐NME) dipeptide, where “XXX” is a placeholder for a three‐letter residue code. *PTM‐Psi* then writes a set of input files and a SLURM script in the current working directory. For example, the code snippet



snc = ptmpsi.protein.Protein()



snc.prepend(chain="A", residue="CYS")



snc.modify("A:CYS1", "nitrosylation")



ptmpsi.nwchem.get_qm_data(snc)




will create a single CYS residue, modify it to SNC, and produce all input files necessary for the first step in the forcefield parameterization according to the following general pipeline: (1) pulls a singularity container with an appropriate version of NWChem, (2) optimizes the geometry and computes the electrostatic potential of both conformations (α‐helix and β‐strand), (3) obtains the Hessian matrix of the optimized conformers, (4) obtains charges, bond, and angle force constants using the generated QM data, and (5) performs a dihedral scan along a given torsion angle. More details can be found in Section 5.

The second part of the pipeline uses the forcebalance method to perform an iterative non‐linear least square fitting to optimize the torsion potentials, using the ForceBalance python package (L. P. Wang et al., 2013, 2014) as the backend. The forcebalance method needs qmdata.txt files generated in the prior step as well as a modified force field with the new residue information. These files can be located in either the current working directory or the paths must be given as an argument.

#### 2.2.4 MD simulations

The *PTM‐Psi* package setups classical MD simulations with GROMACS (Abraham et al., 2015) by generating a batch script that performs a series of steps needed for the initial equilibration of a biomolecular system. These steps include the solvation of the biomolecule, neutralization of the charge, energy minimization, and restrained thermalization using the NVT and NPT ensembles. The restraints are slowly removed during the thermalization process in order to ensure minor perturbation to the original structure. By default, PTM‐Psi will generate a 20 ns unrestrained NPT equilibration step. The user is responsible for checking that the variables to study are correctly equilibrated prior to production data collection.

Many GROMACS options are customizable, including the box type and size, ionic concentration, and the non‐bonded cutoffs. The number of ions needed to neutralize the charge of the protein and to mimic a given salt concentration can also be computed by *PTM‐Psi* using the Screening Layer Tally by Container Average Potential (SLTCAP) method (Schmit et al., 2018). More details about the equilibration strategy used by *PTM‐Psi* are given in Section 5.

#### 2.2.5 Molecular docking

*PTM‐Psi* sets up molecular docking calculations using AutoDock Vina 1.2 (Eberhardt et al., 2021; Trott & Olson, 2010) or Autodock‐GPU packages (Santos‐Martins et al., 2021). If the receptor PDB file does not have the binding site annotated, the user must supply the box parameters to perform the search (box size and center).

*PTM‐Psi* uses the XYZ2MOL package (Y. Kim & Kim, 2015) in order to obtain a Sybyl mol2 file with appropriate atom types and charges, which is subsequently converted into the standard PDBQT file format by means of the Meeko package. The receptor PDBQT file is obtained by processing the PDB file using the *prepare_receptor* script included in the AutoDock Suite.

### 2.3 Force field parameters for *S*‐nitrosocysteine

#### 2.3.1 QM parameterization

To illustrate the utility of *PTM‐Psi*, we present the parameters obtained for SNC following the *PTM‐Psi* pipeline and compare them to other available published values. We generated AMBER FF99SB parameters for most of the cysteine PTMs accessible using *PTM‐Psi* (all parameters available in the Supplementary Material). The only exceptions were CGL and IYY, whose parameters were obtained by analogy with the respective parameters of the GLY, CYX, and GLU residues.

Table 2 lists the partial atomic charges for the SNC atoms not involved in the amide bond. Since we aimed to obtain parameters that were compatible with the FF99SB force field, the *PTM‐Psi* charges were calculated using the *N*‐acetyl‐(*S*‐nitrosocysteinyl)‐*N′*‐methylamine (ACE‐SNC‐NME) dipeptide in the gas phase. The partial charges obtained by Han (Han, 2010) for the FF99SB force field were derived using a similar methodology on the *S*‐nitrosoethanethiol model fragment. Han's methodology also differs from ours in that the geometry optimizations were calculated with a different QM setup. The partial charges obtained by Croitoru et al. (2021). followed the CHARMM36 protocol, which used water interactions, dipole moments, and electrostatic surface potential (ESP) charges as fitting targets and was also derived for the *S*‐nitrosoethanethiol model compound. Despite the differences in the setups, the total charge of the *S*‐nitroso (SNO) moiety is well‐conserved among the three methods. Our method produces more negatively charged S atom (i.e., SG) and more positively charged N atom (i.e., ND) due to the chosen QM density‐functional‐theory level needed to address the higher order oxidation state in the electronic structure (see Section 5.2).

**TABLE 2:** Partial atomic charges used for parameterizing the *S*‐nitrosocysteine residue.

Atom name	*PTM‐psi*	Han (2010)	Croitoru et al. (2021)
CA	−0.0218	−0.1235	0.0700
HA	0.0758	0.0346	0.0900
CB	0.4533	0.2775	0.0500
HB1	−0.0674	−0.0013	0.0900
HB2	−0.0674	−0.0013	0.0900
SG	−0.1959	−0.1384	−0.0230
ND	0.0855	0.0291	−0.1060
OE	−0.1468	−0.1457	−0.1010

*Note*: Important differences due to the quantum‐mechanical method used to obtain the charges are highlighted in gray.

Abbreviation: *PTM‐Psi*, *p*ost‐*t*ranslational *m*odification on *p*rotein *s*tructures and their *i*mpacts.

Table 3 compares equilibrium bond distances, $r_{\mathrm{eq}}$, and harmonic bond stretching force constants, $k_{b}$, or the same three methodologies as those used for determining charges in Table 2. Han's methodology (Han, 2010), which optimizes the structures at the Hartree–Fock level, gives a rather short S—N equilibrium bond distance but an N—O distance of about the same magnitude as the MP2 calculations of Croitoru et al. (2021). In contrast, our calculations with the r^2^SCAN density functional approximation (DFA) enlarge the S—N and shorten the N—O bonds, indicating a less conjugated character. The force constants are similar to those obtained by Croitoru et al. (2021), especially the S—N force constant, which has an unusually low value. The N—O force constant obtained with *PTM‐PSi* reflects the larger double bond character of the bond predicted by the r^2^SCAN DFA.

**TABLE 3:** Equilibrium bond distances and harmonic bond stretching force constants used in different parameterizations of the *S*‐nitrosocysteine residue.

Bond	*PTM‐psi*		Han (2010)		Croitoru et al. (2021)	
	$r_{\mathrm{eq}}$	$k_{b}$	$r_{\mathrm{eq}}$	$k_{b}$	$r_{\mathrm{eq}}$	$k_{b}$
C—S	0.1803	129,555.1			0.1818	165,686.4
S—N	0.1918	79,743.2	0.1656	277,323.9	0.1821	78,240.8
N—O	0.1170	695,768.8	0.1209	660,988.3	0.1217	487,402.5

*Note*: The harmonic potential is defined as $V\left(r\right)=\left(1/2\right)k_{b}{\left(r-r_{\mathrm{eq}}\right)}^{2}$. Distances are given in nm and force constants in kJ mol^−1^ nm^−2^.

Abbreviation: *PTM‐Psi*, *p*ost‐*t*ranslational *m*odification on *p*rotein *s*tructures and their *i*mpacts.

Table 4 lists the equilibrium angles, $\theta_{\mathrm{eq}}$, and the corresponding force constants, $k_{\theta }$. There are large differences in the $k_{\theta }$of the C—S—N and S—N—O angles determined by *PTM‐Psi*. The cause of the difference is not solely due to the electronic structure methodology used to obtain each parameter set. It is also due to the use of the Seminario method (Allen et al., 2018; Seminario, 1996) for deriving harmonic bond and angle force constants directly from the QM Hessian matrix, a method that is known to overestimate angle force constants. *PTM‐Psi* uses the modified Seminario method introduced by Allen et al. (2018). to lessen the stiffness of the angular motions by taking the geometry of the molecule into account. However, the modified Seminario method only corrects force constants of angles that share central atoms (like H—C—S and C—C—S in Table 4). As a consequence, $k_{\theta }$'s of C—S—N and S—N—O angles, which do not share a central atom with any other angle, are likely to still be overestimated.

**TABLE 4:** Equilibrium angles and harmonic angle force constants used in different parameterizations of the *S*‐nitrosocysteine residue.

Angle	*PTM‐psi*		Han (2010)		Croitoru et al. (2021)	
	$\theta_{\mathrm{eq}}$	$k_{\theta }$	$\theta_{\mathrm{eq}}$	$k_{\theta }$	$\theta_{\mathrm{eq}}$	$k_{\theta }$
H—C—S	107.5	293.0	109.5	418.4	111.3	385.8
C—C—S	113.8	487.5	114.7	418.4	112.5	485.3
C—S—N	99.5	1137.0	96.4	551.5	92.3	551.5
S—N—O	117.2	1283.1	115.3	572.4	115.3	642.7

*Note*: The harmonic potential is defined as $V\left(\theta \right)=\left(1/2\right)k_{\theta }{\left(\theta -\theta_{\mathrm{eq}}\right)}^{2}.$ Angles are given in degrees and force constants in kJ mol^−1^ rad^−2^.

Abbreviation: *PTM‐Psi*, *p*ost‐*t*ranslational *m*odification on *p*rotein *s*tructures and their *i*mpacts.

Torsion potential parameters are listed in Table 5. In addition to parameterizing the specific torsion, these parameters compensate for errors introduced by all other parameters in the force field. Interestingly, the torsion potentials obtained using Croitoru et al.'s (2021) methodology and ours are very similar, even though the underlying QM methodologies and overall fitting strategies are different.

**TABLE 5:** Torsion force constants used in different parameterizations of the *S*‐nitrosocysteine residue.

Torsion	*PTM‐psi*			Han (2010)			Croitoru et al. (2021)		
	$\phi_{s}$	$k_{\phi }$	n	$\phi_{s}$	$k_{\phi }$	n	$\phi_{s}$	$k_{\phi }$	n
C—C—S—N	180	0.2399	1	180	5.0626	1	180	0.2100	1
	0	1.4742	2	180	1.6875	3	0	2.8455	2
H—C—S—N	180	1.4613	3	180	5.0626	1	180	1.2024	3
				180	1.6875	3			
C—S—N—O	0	1.0769	1	180	2.0501	1	180	1.6485	1
	180	27.2930	2	180	34.2251	2	180	27.344	2
	180	2.7214	3	180	3.4309	3	180	3.2522	3
	180	0.5033	4				180	2.1694	4
							0	0.4945	6

*Note*: The torsion potential is defined as $V\left(\phi \right)=\sum k_{\phi }\left(1+\cos\left(\mathrm{n\phi}-\phi_{s}\right)\right)$, where n is the multiplicity, and $\phi_{s}$ is the phase. Force constants are given in kJ mol^−1^ and phases in degrees.

Abbreviation: *PTM‐Psi*, *p*ost‐*t*ranslational *m*odification on *p*rotein *s*tructures and their *i*mpacts.

**FIGURE 4:**
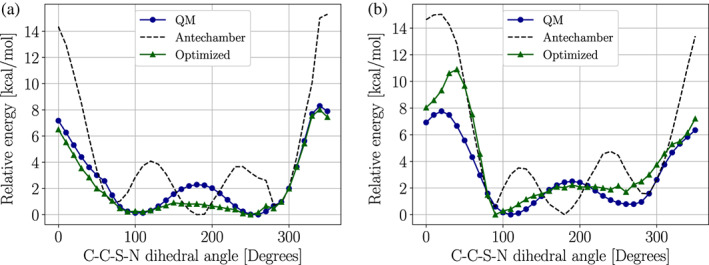
Energy profile obtained by scanning the C—C—S—N dihedral angle of the SNC‐capped dipeptide (ACE‐SNC‐NME) in (a) α‐helix or (b) β‐strand conformations. The dashed line labeled as “Antechamber” was obtained using the general AMBER force field (GAFF2) parameters using the Antechamber program. The green solid line labeled as “Optimized” was obtained using parameters obtained with *PTM‐Psi*. The black solid line labeled as “QM” was obtained using parameters at the density‐functional‐theory level. ACE‐SNC‐NME, *N*‐acetyl‐(*S*‐nitrosocysteinyl)‐*N′*‐methylamine dipeptide; *PTM‐Psi*, *p* ost‐ *t* ranslational *m* odification on *p* rotein *s* tructures and their *i* mpacts.

**FIGURE 5:**
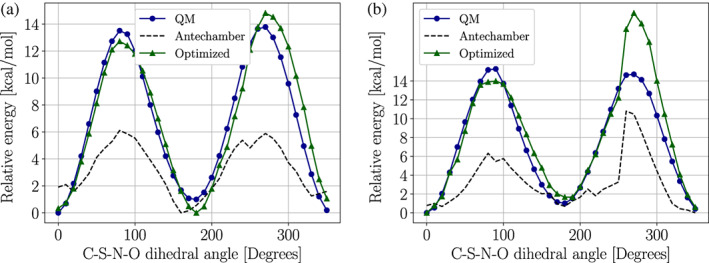
Energy profile obtained by scanning the C—S—N—O dihedral angle of the SNC‐capped dipeptide (ACE‐SNC‐NME) in (a) α‐helix or (b) β‐strand conformations. The dashed line labeled as “Antechamber” was obtained using the general AMBER force field (GAFF2) parameters using the Antechamber program. The green solid line labeled as “Optimized” was obtained using parameters obtained with *PTM‐Psi*. The black solid line labeled as “QM” was obtained using parameters at the density functional theory level. ACE‐SNC‐NME, *N*‐acetyl‐(*S*‐nitrosocysteinyl)‐*N′*‐methylamine dipeptide; *PTM‐Psi*, *p* ost‐ *t* ranslational *m* odification on *p* rotein *s* tructures and their *i* mpacts.

In order to assess the quality of our parameters, we compared dihedral angle scans obtained at the QM and MM levels. Figure 4 shows this comparison for the C—C—S—N torsion in either α‐helix or β‐strand conformations. The α‐helix conformation was notoriously difficult to fit due to overcrowding. However, the largest difference occurs in regions above 6 kcal mol^−1^ from the global minimum. These high‐energy regions are not likely to have a substantial impact on the system dynamics at standard conditions. A similar effect can be seen in the energy profiles obtained by rotating the C—S—N—O dihedral (Figure 5), where larger differences are seen for the α‐helix conformation. Figures 4 and 5 also show the energy profiles obtained by using the parameters given by the Antechamber program using the second‐generation general AMBER forcefield (GAFF2) (J. Wang et al., 2004). The cautionary tale here is that Antechamber parameters might lead to qualitatively different potentials that can affect the *S*‐nitroso moiety dynamics.

#### 2.3.2 Comparison with experimental data

Further validation was obtained by comparing experimental bond distances, angles, and torsions from all structures deposited in the PDB containing at least one *S*‐nitrosocysteine residue in the polymer chain (PDBID, 1BUW, 2CI1, 2HXK, 2IFQ, 2IIY, 2LLT, 2NRM, 2Y33, 2Y34, 3EU0, 3V4N, 4F0H, 4F0K, 4F0M, 4IAH, 4L20, 4L21, 4OO5, 4RKY, 5HBL, 5HBO, 5HBP, 5JJM, 5K95, 5K9G, 5OYA, 6AP9, 6D43, 6NQ5, 6WYC, 6X2E, 6XFT, 7AV7, 7BGI, 7BLX, 7O01, 7R3K, 7VB3, 7VB5, 7VB6).

**FIGURE 6:**
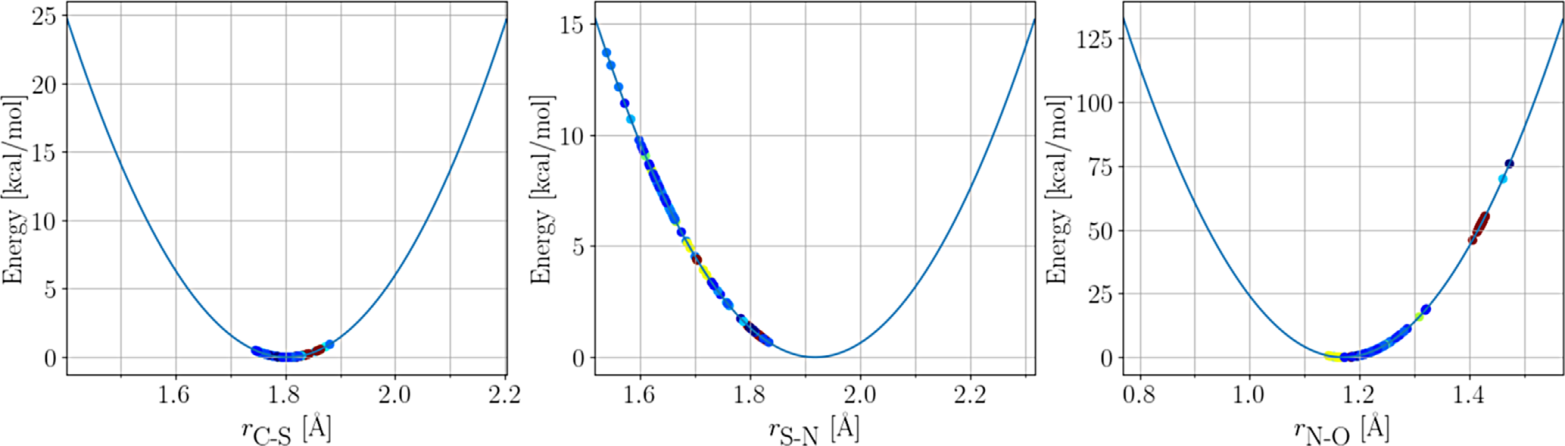
Distribution of the experimental C—S, S—N, and N—O bond lengths (Å) on top of the bond stretching potentials derived in this work. The experimental data are colored by the resolution annotated in the Protein Data Bank.

**FIGURE 7:**
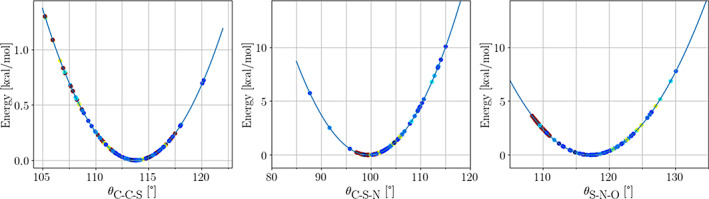
Distribution of the experimental C—C—S, C—S—N, and S—N—O bond angles on top of the angle bending potentials derived in this work. The experimental data is colored by the resolution annotated in the Protein Data Bank.

**Bond lengths and bond angles:** Both lengths and bond angles taken from experiments agree well with our calculations. Figure 6 shows the distribution of the experimental C—S, S—N, and N—O bond lengths plotted on top of the potentials described in the previous section. Each dot represents an experimental value and is colored according to the reported resolution in the PDB file, with the best resolutions represented in blue and the worst resolutions in dark red. The same color code will be used throughout this section. The experimental C—S and N—O bond lengths show a dispersion of around 0.1 Å (excluding data from electron microscopy with poor resolution). The $r_{\mathrm{eq}}$ values used in our parameterizations correlate rather well with the experimental ones. Interestingly, the experimental S—N bond lengths show a wider dispersion of about 0.35 Å and poor agreement with our predicted $r_{\mathrm{eq}}$ of 1.918 Å. Previous theoretical investigations on *S*‐nitrosothiols have also provided rather large fluctuations in the predicted S—N bond length (Baciu & Gauld, 2003; Khomyakov & Timerghazin, 2017; Meyer et al., 2016; Timerghazin et al., 2008), but the best estimate has been placed at around 1.82 Å (Khomyakov & Timerghazin, 2017) for the *S*‐methanethiol model. The difference between the equilibrium bond lengths predicted by the default method built in *PTM‐Psi* and the best theoretical estimate might be due to the simple *S*‐methanethiol model employed by Khomyakov and Timerghazin (2017), as it lacks many intra‐ and inter‐residue interactions of our ACE‐SNC‐NME dipeptide. In light of these results, it seems plausible that the experimental data show systematic errors toward short S—N bonds and long N—O bonds. Figure 7 shows the comparison between the experimental bond angles and the bending potentials of our forcefield parameterization. In this case, most of the experimental observations are located within 5° from the bottom of each well, although there are some outliers in every case.

**FIGURE 8:**
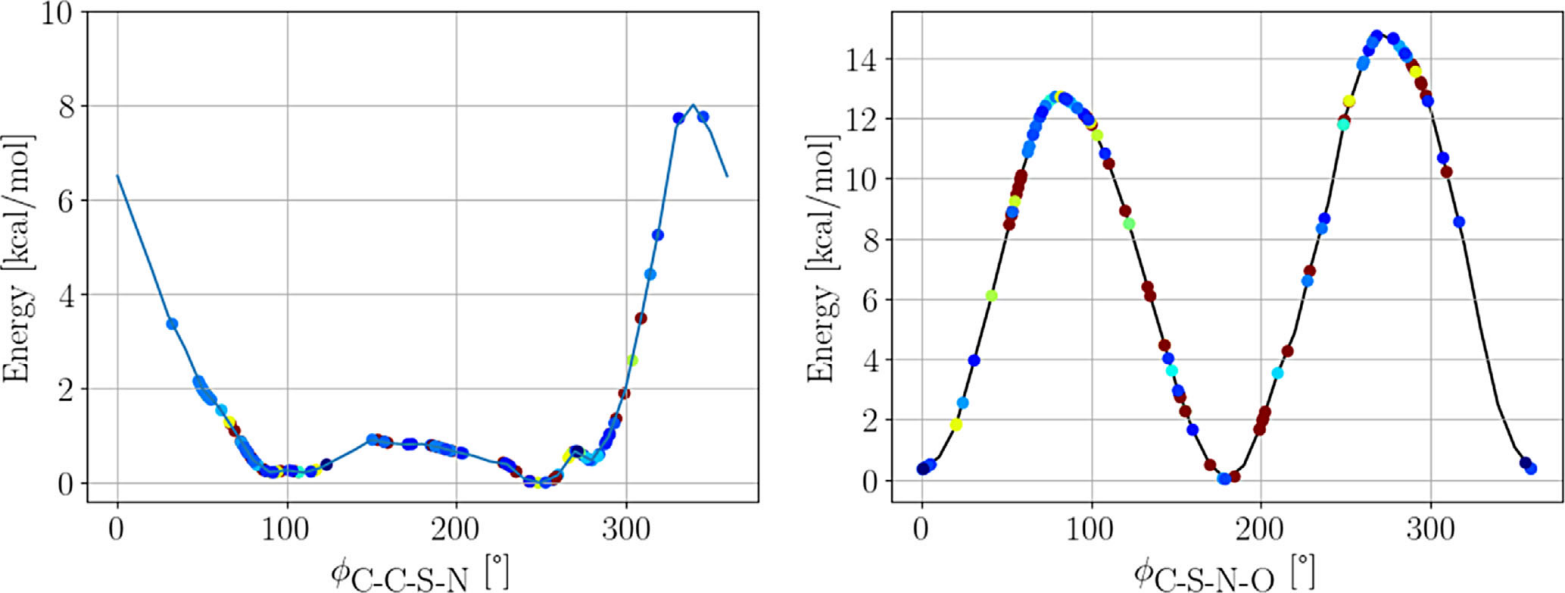
Distribution of the experimental C—C—S—N and C—S—N—O dihedral angles on top of the potential energy surfaces generated by scanning the corresponding dihedrals. The experimental data is colored by the resolution annotated in the Protein Data Bank.

**Dihedrals:** There is also a very good agreement between the experimental C—C—S—N torsions and the energy profiles generated for the α‐helix and β‐strand conformations of the ACE‐SNC‐NME dipeptide. The left panel of Figure 8 shows that most of the experimental data lie around the minima of the energy profile for the α‐helix conformer. Similar observations follow for the β‐strand conformer, as the location of the minima and maxima remains the same for both conformers (see Figure 4).

Interestingly, we observed that the experimental C—S—N—O torsional angles disperse across nearly all possible values (right panel of Figure 8), with about 50% of the data closer to the maxima of the energy profiles of both conformers (regardless of the quality of the experiment). We speculate that the widespread of the experimental values for this torsional angle are due to the non‐bonded interactions, including steric effects and hydrogen bonding, between the SNO moiety and the rest of the corresponding protein. Note that the C—S—N—O torsion is the one most likely to change as a response to environmental interactions because it is the terminal torsion of the side chain, and its change involves fewer atomic movements. In other words, a change of the C—S—N—O torsion requires less work as compared to a change of the C—C—S—N or other internal torsions. Lastly, we would like to emphasize that none of these environmental effects are captured in the gas‐phase energy profile of the dipeptide.

Overall, the experimental crystallographic data can be reproduced reasonably well by using the parameters obtained with the default pipeline of PTM‐Psi, even with a challenging electronic structure at hand, as is the case of the *S*‐nitrosothiol moiety.

### 2.4 Free energy perturbation of *S*‐nitrosylated GAP2 protein

**FIGURE 9:**
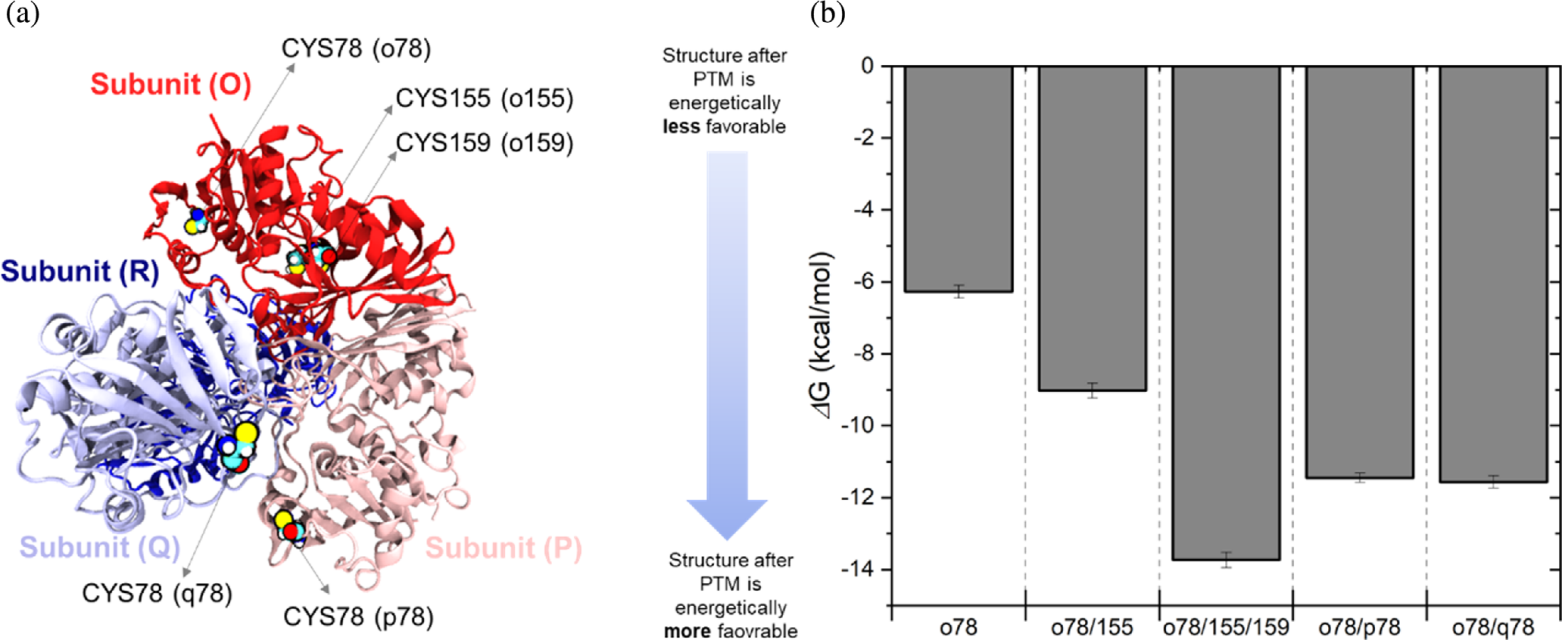
(a) A representative crystal structure of GAP2 from *Synechococcus elongatus PCC 7942* with selected cysteines residues for modifications highlighted by the van der Waals surface model (yellow, cyan, white, blue, and red colored atoms represent sulfur, carbon, hydrogen, nitrogen, and oxygen atoms, respectively). GAP2 is a homo‐tetramer, and each subunit is highlighted by a different color. (b) Free energetic differences between the protein before and after nitrosylations of selected cysteine(s) where the lower Δ*G* indicates that the protein after PTM is energetically more favorable. PTM, post‐translational modification.

This corresponds to Scenario I in the use case study to investigate the impact of strategically PTMed cysteines on the stability of GAP2 that includes P, Q, R, and S subunits using the free energy perturbation (FEP) approach. We consider GAP2 subunit O to be in a catalytic active state (where Cys155 is not PTMed) as well as an inactive state where Cys155 is PTMed. Other cysteine sites of 78 or 159 are strategically PTMed in the GAP2 complex, as shown in Figure 9a. We investigated five cases that involve CYS78, CYS155, and CYS159 in subunit O, and CYS78 in subunits P and Q considered for modifications: (1) nitrosylations of CYS78 (o78); (2) nitrosylations of CYS78 and CYS155 in subunit O (o78/155); (3) nitrosylations of CYS78, CYS155, and CYS159 in subunit O (o/78/155/159); (4) nitrosylations of CYS78 in subunit O and subunit P (o78/p78); and (5) nitrosylations of CYS78 in subunit O and subunit Q (o78/q78).

Our FEP calculations showed that PTMed CYS lowers the stability (Δ*G* < 0) in all five cases in Figure 9b, signifying that nitrosylated cysteines tend to favor the local environment compared to cysteines before modifications. Specifically, o78/155/159 showed the lowest ΔG. This highlights that the structure after the nitrosylation of o78/155/159 is energetically the most stable among all tested cases. Considering the location of cysteines adjacent to catalytic residues (CYS155) that could sterically affect protein functions, o78/155/159 could be the first candidate for further experimental studies. We evaluate the changes in the local structures among the five cases in the section that follows.

### 2.5 GAP2 structural changes due to S‐nitrosylation of the GAP2 protein

**FIGURE 10:**
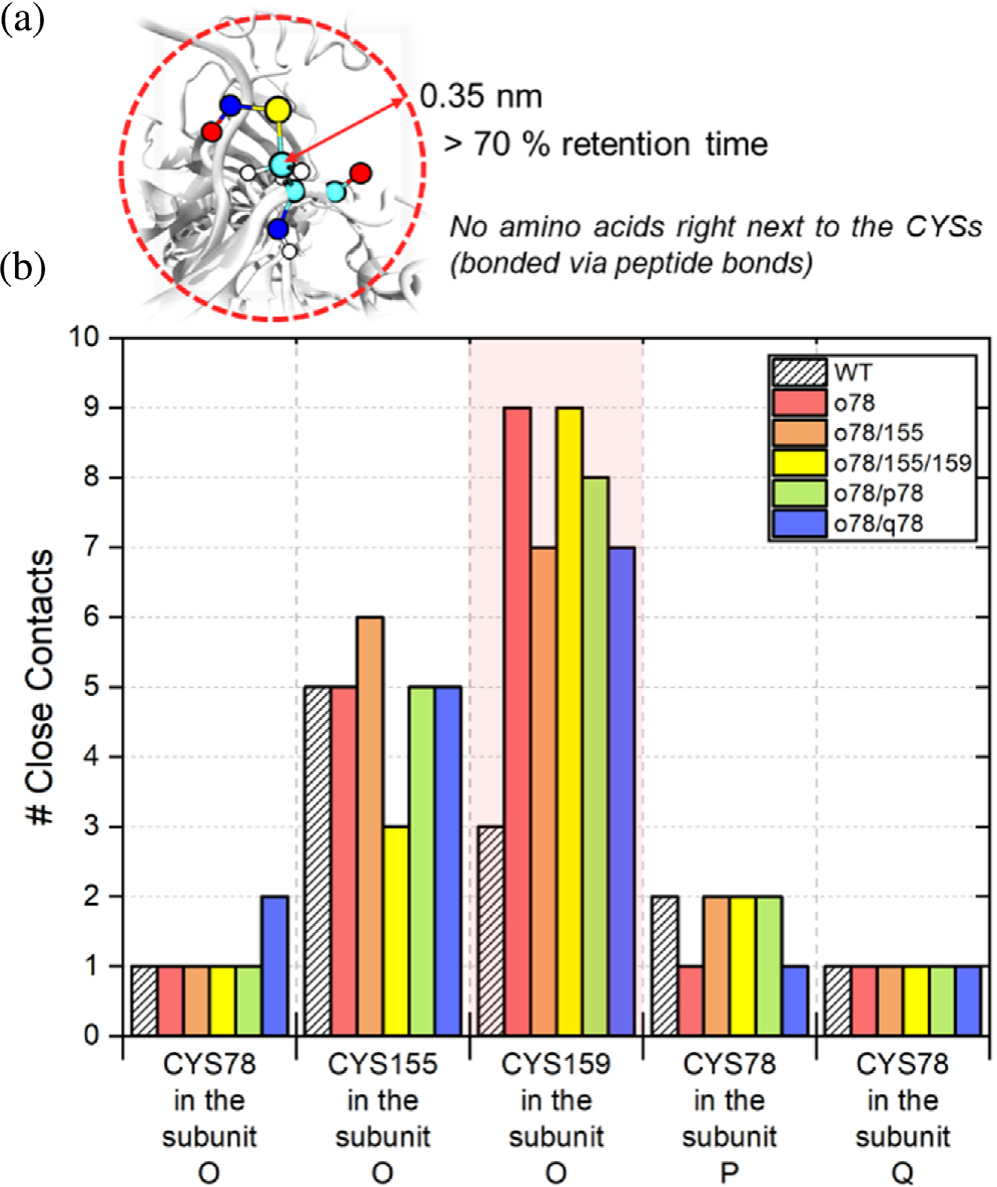
The number of non‐bonded close contacts affected by selected PTMed cysteines against the wild type (WT). (a) Schematic snapshots illustrating definitions of close contact used in this study. (b) The number of adjacent amino acids that make close contact with selected CYSs. Red, orange, yellow, green, and blue bars represent the individual cases after modifications of CYS at o78, o78/155, o78/155/159, o78/p78, and o78/q78, respectively. The white bars with a pattern represent the WT without PTMs. The light shade in red showed that CYS159 in the subunit O reveals the most changes in the number of close contacts after PTMs. PTM, post‐translational modification.

We analyzed the number of close non‐bonded contacts (Figure 10a) with selected *S*‐nitrosocysteine from the FEP simulations in Figure 9b. In Figure 10b, when compared with the wild‐type (WT) GAP2 (without PTMs), nitrosylations on cysteines altered the interactions with adjacent amino acids, accounting for changes in close non‐bonded contacts to a different extent. CYS78 is located at the orifice of the catalytic site and near the surface of GAP2. PTM at this cysteine does not significantly change the overall close contacts. However, the number of amino acids that make close contact with *S*‐nitrosocysteine at 159 in subunit O significantly increases from 3 to 7–9 (shaded in red in Figure 10b), contributing to the most energetically favored structure after PTM in Figure 9b.

**FIGURE 11:**
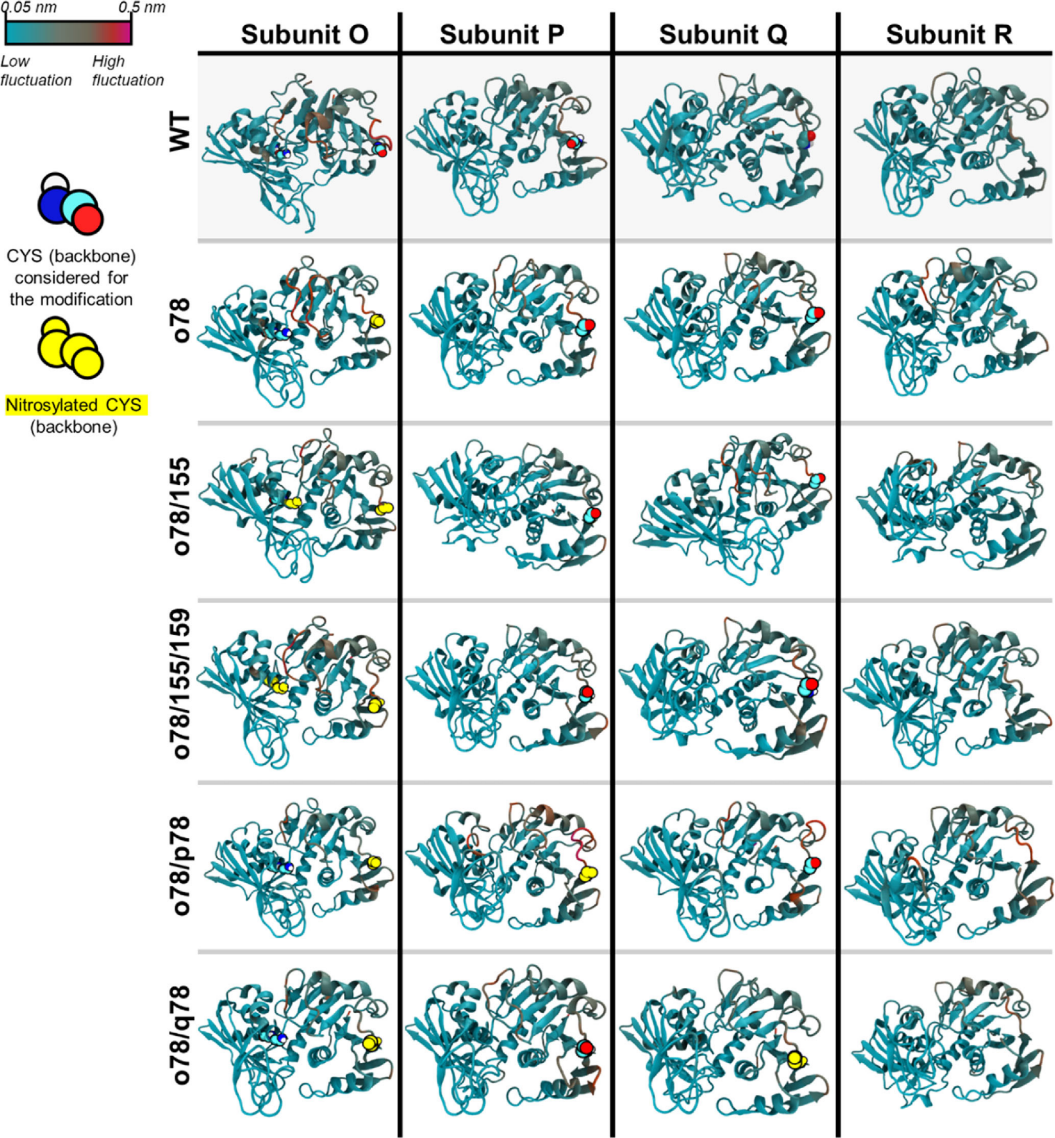
Representative snapshots of each subunit of GAP2 and its modified subunits using a Cartoon drawing model. The flexibility of the amino acids is calculated via root mean square fluctuations (RMSFs) and highlighted by different colors ranging from cyan (low fluctuated amino acids) to red (high fluctuated amino acids). CYSs considered for the modifications are represented by larger van der Waals (vdW) surface models, and nitrosylated CYSs are represented by yellow vdW surface models.

We next evaluated whether these PTMs alter the secondary or tertiary structures of GAP2. This includes the regions near catalytic residues by examining the changes in fluctuations of GAP2 with respect to the crystal structures in Figure 11. When compared to the root‐mean square fluctuation (RMSF) from the WT case, PTMs in all five cases we investigated do not significantly change the overall RMSFs. CYS78 is located at the protein's periphery. The regions in subunits O, P, and Q near CYS78 are inherently flexible. Furthermore, the results showed that the regions near CYS155 and CYS159 in subunit O are also relatively flexible in the WT. PTMs at these two cysteines do not introduce additional flexibility to that region. Also, the secondary structure analyses in Figure S1 showed that PTMs marginally affect the secondary structures of GAP2. This indicates that nitrosylations affect local contact formation, which leads to changes in their interactions with adjacent residues. However, these modifications do not significantly affect protein secondary and tertiary structures and dynamics.

### 2.6 The impact of *S*‐nitrosylation on the GAP2‐ligand binding affinity

**FIGURE 12:**
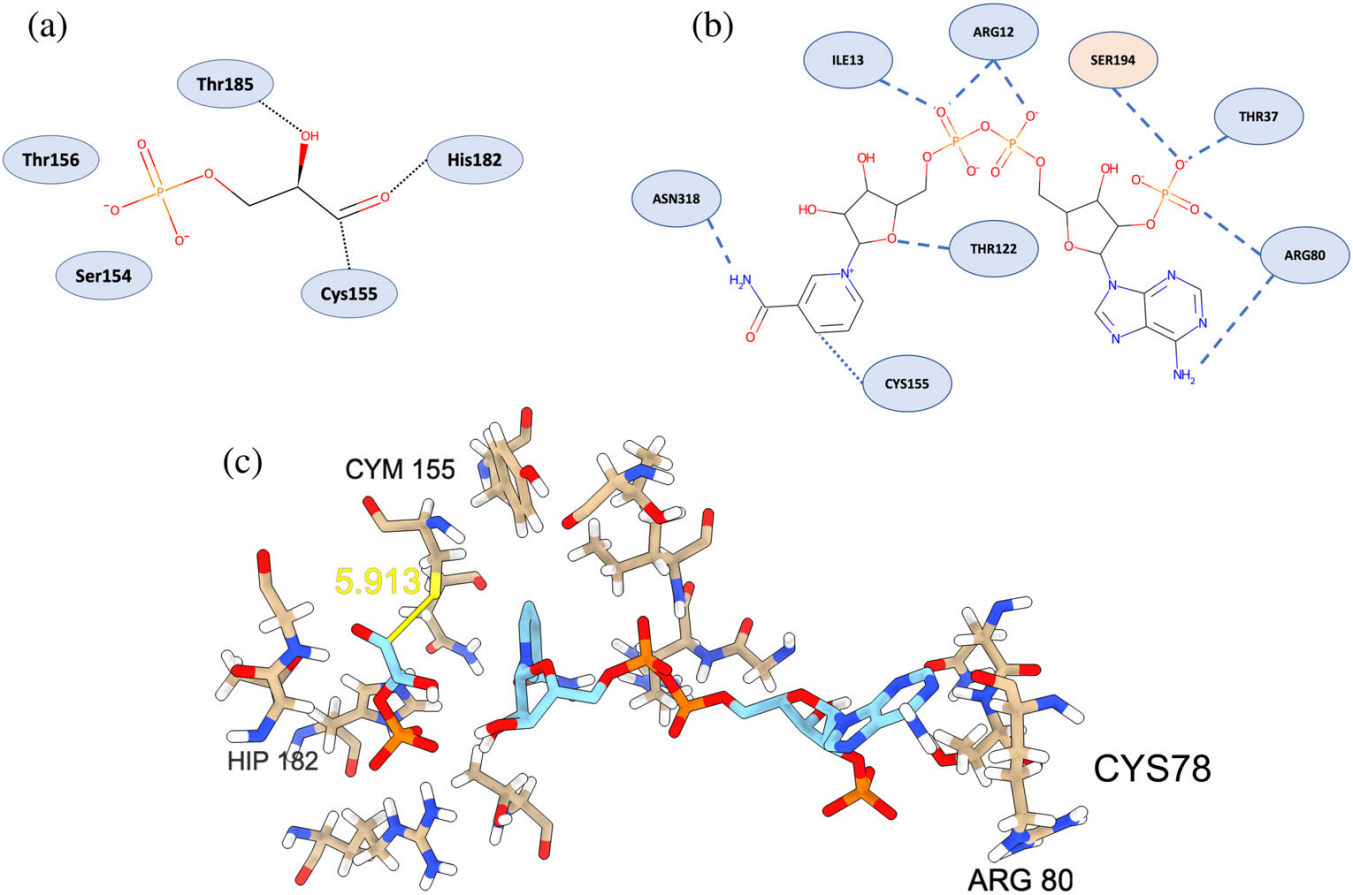
Two‐dimensional interaction map of (a) GAP2 and GA3P and (b) GAP2 and NADP. The single residue in the red background corresponds to an adjacent protein chain, whereas the rest of the residues belong to the same chain as the NADP molecule. (c) GA3P and NADP+ docked into the GAP2 wild‐type active site. Carbon atoms from the ligands are shown in light blue, and from the protein in light brown. CYM represents deprotonated cysteine. HIP represents doubly protonated histidine. A proton was transferred from Cys to His in order to have the right docked structures for a Michaelis complex in the wild type. All residues shown correspond to the binding site listed in the Q55245 entry of the UniProt Knowledge Base. CYS78 was not part of the Michaelis complex but is labeled to show its proximity to ARG80. NADP, nicotinamide adenine dinucleotide phosphate.

We next moved to Scenario (II) in the use case and compared the impact of selected PTMed cysteines on the ligand‐binding of GAP2 that binds two ligands—glyceraldehyde‐3‐phosphate (GA3P) and/or NADP^+^—by employing molecular docking simulations on the WT and several PTMed subunits O of the GAP2 protein. To this end, both GA3P and NADP^+^ were docked into the catalytic site of GAP2 subunit O. The expected contacts between the GA3P substrate and GAP2 participating in chemical reactions at the catalytic site are shown in Figure 12a. The catalytic active site of GAP2 contains the residue CYS155 (not PTMed) as the nucleophilic site that attacks the GA3P carbonyl, while His182 facilitates the CYS‐SH deprotonation and helps to hold the carbonyl group in place.

The expected contacts between the GAP2 and the NADP^+^ are shown in Figure 12b. Residue CYS155 does not react directly with the NADP^+^ cofactor. However, the nicotinamide moiety from NADP^+^ must remain near the hemithioacetal formed after the CYS‐S^−^ nucleophilic attack. Another important feature observed in NADP^+^‐dependent enzymes is that the adenine ring, far away from CYS155, faces the side chain of ARG80 and forms hydrogen bonds with it. ARG80 residue also forms hydrogen bonds with the 2′‐phosphate group of a neighboring ribose (Carugo & Argos, 1997). The same 2′‐phosphate group is also involved in hydrogen bonds with residues THR37 of the same subunit and with SER194 of an adjacent chain. These interactions seem to play a crucial role in the discrimination between NAD^+^ and NADP^+^ (Carugo & Argos, 1997; Kitatani et al., 2006) binding.

Interestingly, no scoring function (vina, vinardo, or ad4) of Autodock Vina could re‐dock GA3P or NADP^+^ by themselves to the catalytic site of the WT enzyme. To achieve the chemical interactions depicted in Figure 12a,b, we propose that the proton of the CYS155 thiol group is transferred to HIS182 prior to the docking simulation. The resulting negatively charged cysteine is labeled as CYM155 in Figure 12, whereas the positively charged histidine is labeled as HIP182 in the same figure. After the proton transfer, both GA3P and NADP^+^ are docked to GAP2 concurrently using the “multiple‐ligand” docking strategy of Autodock Vina. The estimated binding energy obtained with the *vina* scoring function lies around 10.7 kcal mol^−1^ (see Table 6) for a Michaelis complex in the WT. We also repeated the docking simulations in the PTMed GAP2 using a similar strategy with the caveat that the *S*‐nitrosocysteine residue in position 155, whenever present, remains neutral since there are no protons in the *S*‐nitroso group. The PTM of targeted cysteines (e.g., CYS159 or CYS78) has both electrostatic and steric effects that affect the binding affinity towards GA3P and NADP^+^, even when the modification occurs only at the most remote cysteine position (CYS78). The induced locomotions made bi‐ligand docking on GAP2 worse than that of the WT. The best docking scores showing the correct binding position for NADP^+^ are listed in Table 5. Note that *S*‐nitrosylation of CYS78 did not lead to the Michaelis complex shown in Figure 9c; thus, the “not available (NA)” result in Table 5.

**TABLE 6:** Docking scores (binding potentials in kcal mol^−1^) of conformations showing the expected contacts between GAP2 and the ligands GA3P and NADP^+^.

GAP2 form	Score
WT	−10.7
O78	NA
O78/155	−9.0
O78/155/159	−10.0

*Note*: WT refers to the wild‐type protein, while others contain *S*‐nitrosocysteine in the numbered positions of subunit O. The score shows an NA when a Michaelis complex could be found among the top 100 docked candidates.

### 2.7 Allosteric regulation through PTM‐induced conformational changes

**FIGURE 13:**
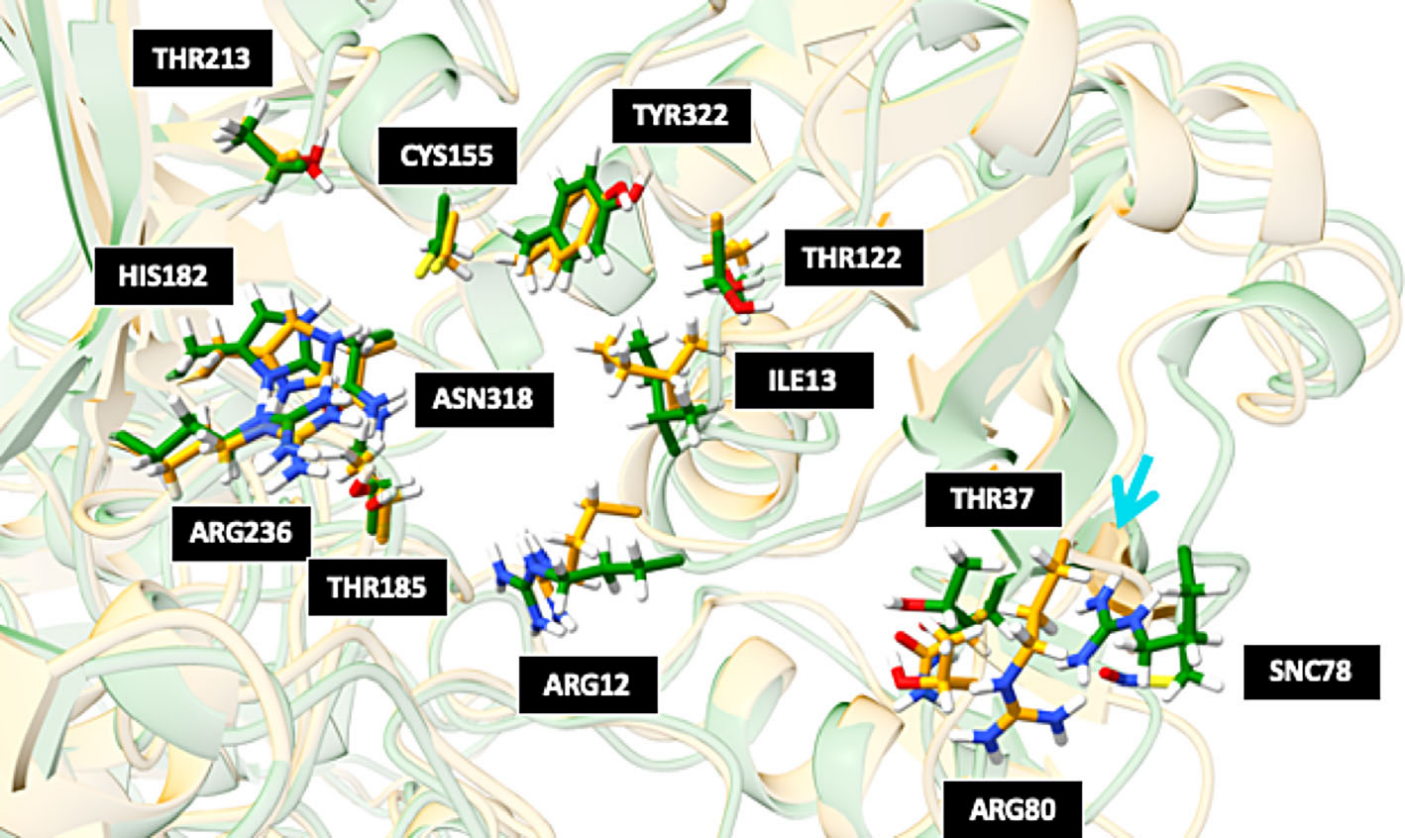
Comparison of the differences in the subunit O catalytic sites between GAP2 wild‐type (orange) and the nitrosylated enzyme in position Cys78 (o78, green). For clarity, the structures of the two ligands are not shown. Most of the side chains display minor movements, except for those in the lower right corner pointed to by a cyan arrow: a hydrogen bond formed between *S*‐nitrosocysteine in Position 78 and arginine in Position 80 disturbs the interaction between ligands and the side chains of GAP2 affect the binding of NADP^+^. NADP, nicotinamide adenine dinucleotide phosphate.

In Table 6, we noticed that the *S*‐nitrosylation of CYS78 in subunit O (i.e., o78) significantly impeded the docking of bi‐ligands (GA3P and NADP^+^) at the catalytic site with a score of “NA”. Although CYS78 resides near the orifice of the catalytic binding site without being involved in the chemical reaction, it is intriguing to ask how its *S*‐nitrosylation affects bi‐ligand docking. With further structural analysis, we discovered that the *S*‐nitroso group of CYS78 forms a weak hydrogen bond with the side chain of ARG80, a key player in the binding of NADP^+’^ through its interaction with the adenine. This hydrogen bond alters the position of the guanidinium moiety of ARG80 (see Figure 13) and impedes its interaction with NADP^+^. Since the cofactor could not bind to the active site, it is likely that *S*‐nitrosylation at CYS78 remotely affects the chemical reaction. This leads to a novel mode of interaction remotely managing the dehydrogenation reaction. PTMs at the cysteines create novel mechanisms of allosteric inhibition of GAP2, in which the overall conformational changes in the protein structures are minimal, while the chemical modification of cysteine side chains near the protein surface responsive to environmental changes creates an additional knob to “turn on” or “turn off” chemical reactions inside the catalytic site remotely. This hypothesis could be tested experimentally in future studies.

### 2.8 Experimental analysis of the PTMed cysteines on the GAP2 protein

One objective of developing the utility of *PTM‐Psi* is to provide structural insights into PTM in the redox proteomics data. To elucidate its utility, we profiled the cysteine oxidation in cultured *S. elongatus* PCC 7942 using an established MS protocol (see Section 5). After the initial blocking of free thiols, all oxidized cysteine thiols were reduced and captured by thiol‐affinity resin and were subjected to Liquid Chromatography with tandem MS (LC–MS/MS) analysis. The annotation of oxidation on the GAP2 is provided in Table 7. Notably, CYS78, CYS155, and CYS159 of GAP2 were all observed to be oxidized in cells. The site occupancy (i.e., oxidized thiols vs. total thiols at individual sites) of CYS78 was approximately 13.4%, suggesting PTMed CYS78 under a physiological condition. CYS155 and CYS159 were either oxidized individually or concurrently in the experiments. *S*‐nitrosylation (SNO), *S*‐glutathionylation, and disulfide (S—S) were possibilities for the thiol oxidations at these cysteine sites. In addition, there may be many combinatorial ways of modification. It is difficult to pinpoint specific PTMs on the exact cysteines based on only redox proteomic data.

**TABLE 7:** Profiling of oxidized cysteine sites of GAP2 in *Synechococcus elongatus* PCC 7942 (gene: *gap2*, UniProt ID# Q9R6W2_SYNE7).

Cys ID	Representative sequence^a^	PSM^b^
CYS78	K.IVC@DRNPLNLPWK.E	20
CYS155 and CYS159	R.HEDFAVISNASC@TTNC@LAPVAK.V	74
CYS155	R.HEDFAVISNASC@TTNC#LAPVAK.V	40
CYS159	R.HEDFAVISNASC#TTNC@LAPVAK.V	45

^a^ Representative sequence of identified cysteine‐containing peptides. @ indicates oxidized cysteines that were reduced, captured, and alkylated with iodoacetamide. # shows reduced cysteines that were initially blocked by N‐ethylmaleimide.

^b^ Peptide spectra match (PSM), that is, the number of spectra matched to a given peptide sequence.

## 3 DISCUSSION

### 3.1 *PTM‐Psi* workflow complements redox proteomics experiments

Our work extended the investigation of PTMs on a protein's structures and binding properties, extending the research scopes that were typically quite limited to a few atoms because of the computationally prohibitive cost. Take GAP2, for example; the investigation of binding with one of the two ligands was conducted using a QM approach, followed by a short run of MD (Araújo Silva et al., 2012; Reis et al., 2013). The chemical activities were limited to the electronic structures of a few atoms within the radius of a few angstroms surrounding an active site. Due to the expensive computational cost, only the ligand and part of the sidechains of the protein were treated quantum mechanically, while the remainder of the structure was represented by MM. To investigate the MD of protein beyond a few atoms, a short MD approach was employed to inspect the motion of cysteine residues near the active sites of GAP2 (Hyslop & Chaney, 2022) or conformational changes upon ligand‐binding (da Costa et al., 2021). However, a comprehensive investigation of PTM's impact on the protein dynamics still remains to be done.

Motivated by the need for a workflow to comprehensively provide insights into the structures and dynamics in the redox proteome data derived from MS (Table 7), we developed a suite of tools to elucidate the impact of oxidated cysteines on the structure and function of proteins without overburdening the computational cost and human endeavor. *PTM‐Psi* streamlines the first step of structural inference from sequences, modification of the PTMed residues, and the parameterization of the force field for non‐standardized amino acids. For the latter, the tool generates the initial configurations in an AMBER format and can be visualized by ChimeraX (Pettersen et al., 2021). The force fields for thiol‐cysteines were developed using a QM theory at the level that was proven to correlate with experiments (Qin et al., 2020). A user can then use these initial configurations and force field parameters for the ensuing calculation of the FEP on the protein structure based on MD or for ligand docking. Although we used GAP2 as an example, this custom‐designed workflow can be scaled up to the entire redox proteome with automation as the next step when extensive computational resources become readily available. The FEP on each thiol cysteine site at a proteomic scale can offer the needed thermodynamics parameters in the systems modeling of regulating redox‐related functions (Li et al., 2020; Su et al., 2017) as future directions.

### 3.2 *PTM‐Psi* reveals possible impacts of intermediate oxidation cysteines on GAP2

A breadth of research has been conducted to track the shifts in the redox homeostasis of cyanobacteria in response to oxidative stress (Ansong et al., 2014; Guo et al., 2014; Lindahl & Kieselbach, 2009; Sadler et al., 2014). A study by Ansong et al. (2014). found that light‐to‐dark transitions significantly shifted the redox status of more than 300 proteins, including GAP2, in *Synechococcus* sp. PCC 7002. At least one of the protein's Cys residues went from oxidized to reduced status in the majority of the proteins. The cysteine thiols are subjected to redox‐dependent PTMs as intermediate oxidation states (Mattioli et al., 2022). Glutathione and disulfide on cysteines at catalysis sites are shown to be the end‐product of the oxidation (Zaffagnini et al., 2019), and the structures of these oxidation states have been shown to significantly alter the GAP2 structures and even form insoluble aggregates.

We chose SNO as a model to explore the intermediate oxidation cysteines on GAP2. While SNO is transient and labile, it provides clues to the incremental structural changes (Mattioli et al., 2022) in the midst of the oxidation process. The investigation of bulky glutathione or disulfide bonds will be addressed in future work because they both induce significant conformational changes in GAP2, and a simple FEP tool may not be efficient.

### 3.3 GAP2 resembles a “puzzle box” with a hidden mechanism and intricate locking mechanisms that can be remotely activated by PTMed cysteines

In recent decades, GAPDH has been reported to be one of the most prominent cellular targets of PTMs and sensitive to redox‐dependent regulation (Tossounian et al., 2020). Multiple types of redox PTMs formed on GAPDH catalytic cysteines led to alteration of conformations, enzymatic activity, subcellular localization, and interaction with other proteins (Tossounian et al., 2020). However, only limited evidence can be found about the redox states and PTMs of photosynthetic GAPDH (i.e., GAP2) in cyanobacteria, which plays a critical role in carbon fixation. In this study, we chose three cysteines (Cys78, Cys155, Cys159) from GAP2 as targets of redox PTMs to investigate the impact of redox PTMs on GAP2 and possible underlying mechanisms.

For GAPDH, CYS155 and CYS159 from the evolutionarily CTTNC motif form a disulfide bond at the catalytic site in many species, but that is not the case for the GAP2 from cyanobacteria (McFarlane et al., 2019). CYS78 is located at the protein surface and is not evolutionarily conserved like the other two cysteines. We showed that the oxidated modification of the three cysteines that are near the two ligands in GAP influences the movement of the ligands. We speculated that the PTMed CYS78 may influence ARG81, which forms hydrogen bonding with NADP (McFarlane et al., 2019). PTMed cysteines might remotely influence the catalytic site at CYS155 through the movement of the two ligands over distance. We speculated that the GAP2 protein resembles a “puzzle box” with an exposed cysteine serving as a switch. Although the overall structural dynamics do not correlate with PTMs (Figure 11), PTMed side chains affect the alignment of ligands through their local contacts. PTMs act as sensors that intricately switch on hidden catalytic sites through the connection of the two ligands (Figure 13). Since CYS78 is unique to our cyanobacteria strain and can influence the movement of ARG81, we speculate that its oxidation plays a critical role in the regulation of light‐dependent catalytic reaction through the formation of protein complexes with GAP2 and other enzymes in the Calvin–Benson cycle (Mattioli et al., 2022; McFarlane et al., 2019).

## 4 CONCLUSION

Here, we provide an integrative approach to understanding the molecular basis of PTMs that underlie the regulation of the protein functions. Our workflow is one of the first puzzle pieces, after the era of AlphaFold, to help understand the dynamics of the protein interactomes that are often regulated by PTM in response to environmental changes. We do not expect our work will be in agreement with every experiment. If necessary, users can tune their own force fields using a different level of QM theories. However, this workflow opens the door for uncovering molecular insights based on redox proteomics datasets that can enable the development of novel molecular hypotheses. More work remains to be done to integrate experiments with computational modeling—especially when the system of interest expands into a proteomics scale where computational resources are readily available—as the complexity and the challenges of interpreting the effects of PTMs on biological functions continue to increase.

## 5 MATERIALS AND METHODS

### 5.1 Case use study: Oxidation of thiol side chain of GAP2

We took cyanobacterial NADP‐dependent GADPH (also known as homotetramer NADP‐GADPH protein from *Gap2* gene; Kitatani et al., 2006) of *S. elongatus PCC 7942* as an example. This GADPH, one of the key enzymes from the Calvin‐Benson cycle, is unique to photosynthetic fixation of CO_2_. Therefore, in our study, we referred to it as “GAP2”—a redox‐sensitive enzyme—to distinguish it from other GADPH homologous proteins. GAP2 catalyzes the interconversion between 1,3‐bisphosphoglycerate and d‐glyceraldehyde‐3‐phosphate in the Calvin–Benson cycle (Koksharova et al., 1998). GAPDHs have two anion‐binding sites, the *P*_s_ (substrate phosphate ion site) and the *P*_i_ (inorganic phosphate ion site), near the catalytic CYS155 residue, and they are important to enzymatic reactions. Its enzymatic function is regulated by the cellular redox state that governs its redox PTMs on cysteines (Michelet et al., 2013) and their reaction with metabolites. Two scenarios were used to illustrate the expected outcome of using *PTM‐Psi* to investigate the PTMs on CYS78, CYS155, and/or CYS159 on the subunit O of GAP2, PDBID: PDBID, 2D2I: (1) the impact of PTMs on protein stability using FEP and (2) the impact of PTMs on the binding of GAP2's two ligands.

### 5.2 Experimental characterization of PTM CYS

A culture of *S. elongatus* PCC 7942 cscB/SPS (Li et al., 2017) was prepared by transferring exponentially growing cells into 1‐L Roux bottles containing 500 mL of modified BG‐11 medium to have an initial cell density of 2.5 × 10^7^ cells mL^−1^. BG11 medium was supplemented with 0.09 g L^−1^ of Yeast Nitrogen Base without amino acids and ammonium sulfate (H26271.36, Thermo Fisher Scientific Inc., USA), 0.264 g L^−1^ of (NH_4_)_2_SO_4_ (J64180.A1, Thermo Fisher Scientific Inc., USA), 0.174 g L^−1^ of K_2_HPO_4_, (60,356, Sigma‐Aldrich, USA), and 1 mM of isopropyl‐β‐d‐thiogalactopyranoside (I56000, RPI, USA). Flasks were incubated at 30 ± 1°C, mixed with a 1″ octagon spin bar at ~200 rpm, supplied with filter‐sterilized N_2_ that was enriched with CO_2_ to 1% (v/v), and continuously illuminated by an LED Grow Light to provide a light intensity of ~650 μmol m^−2^ s^−1^ (Monios‐L full‐spectrum sunlight replacement at 400–800 nm, 5000 K). *Light condition cell harvesting*: on the sixth day (or after 143 h), 10 mL aliquots (OD750 nm of ~1.14) from each flask were transferred to 15‐mL conical Falcon tubes and centrifuged at 4700*g*, 4°C, for 5 min to pellet cells. Supernatants were removed, and cell pellets were flash‐frozen with liquid N_2_ and stored at −80°C prior to protein extraction. *Dark condition cell harvesting*: the remaining culture was transitioned to dark condition (lights off and Roux bottles covered in two layers of aluminum foil) for 2 h. At harvest time, lights were kept off, and sample tube racks were covered in foil to minimize light exposure. All other sample harvesting and processing details are the same as those for the light condition.

Samples were processed as previously described (Guo et al., 2014). Briefly, cell pellets were resuspended and incubated with 10% trichloroacetic acid on ice to preserve the redox proteome and partially lyse the cells. Then, lysis buffer containing 100 mM *N*‐ethylmaleimide (NEM), 250 mM (4‐(2‐hydroxyethyl)‐1‐piperazineethanesulfonic acid) (HEPES) pH 7.5, 10 mM ethylenediaminetetraacetic acid (EDTA), 0.5% (w/v) sodium dodecyl sulfate (SDS), and 8 M urea was added to cell pellets, followed by brief sonication and incubation at 37°C for 2 h. Bead beating was conducted to extract more protein. Then, the protein was precipitated in acetone overnight at −20°C. The resulting protein samples were reduced with dithiothreitol and subjected to a resin‐assisted capture (RAC) protocol to specifically enrich oxidized cysteines in the proteome. The detailed experimental procedure of RAC is described elsewhere (Guo et al., 2014). The enriched samples were analyzed by a nanoAcquity UPLC system (Waters) coupled to a Q‐Exactive HF‐X Orbitrap Mass Spectrometer (Thermo Scientific, San Jose, CA). A 120‐min LC gradient was used. A full MS scan was acquired over the range of *m*/*z* 400–1800. MS/MS was conducted in a data‐dependent mode (DDA). LC–MS/MS raw data were searched against the *S. elongatus* PCC 7942 UniProt database (downloaded on March 8, 2022) using MS‐GF+. The spectral level false discovery rate was controlled at ≤1% based on a target‐decoy searching strategy. Data analysis was performed by R.

### 5.3 Force field parameterization for nonstandard cysteine amino acid

We used *S*‐nitrosylation on the side‐chain group of cysteine as an example to parametrize force fields using QM data. We carried out these QM calculations with NWChem using the ACE‐SNC‐NME dipeptide and derived the molecular mechanics (MM) force field parameters for the entire system by geometrically optimizing the bond lengths, bond angles, and dihedral angles.

Step (1): Force field parameters for the non‐standard cysteine residues were obtained using the ACE‐SNC‐NME dipeptide in both the ⍺‐helix (*φ*, ψ = −60°, −45°) and the β‐strand (*φ*, ψ = −135°, 135°) conformations. These conformers were pre‐optimized using the semiempirical GFN2‐xTB method (Bannwarth et al., 2019) recently interfaced to NWChem. The structures were then optimized within Kohn‐Sham Density Functional Theory using the r^2^SCAN‐D3(BJ) (Ehlert et al., 2021; Furness et al., 2020; Grimme et al., 2010, 2011) exchange‐correlation DFA and the robust density fitting technique (Dunlap et al., 1979; Whitten, 1973).

The def2‐TZVP (Weigend & Ahlrichs, 2005) orbital basis and the def2‐universal‐jfit (Weigend, 2006) Coulomb fitting basis set was used along with a Lebedev (Lebedev, 1976; Lebedev & Skorokhodov, 1992) numerical integration grid with 120 radial shells and angular momentum order 41. During the optimizations, the *φ*/ψ torsion angles were kept fixed to the starting values. *PTM‐Psi* allows users to override the default settings described above to choose any exchange‐correlation DFA, orbital and fitting bases, numerical integration grids, and other convergence stabilization options available in NWChem. This theoretical setup has proven to be robust enough for the accurate description of the thermochemistry and kinetics of compounds of main group elements, as well as the complex chemistries seen in many transition metal complexes (Ehlert et al., 2021; Furness et al., 2020; Grimme et al., 2010, 2011).

Step (2): Multiconformational RESP partial charges were then generated for the non‐standard cysteine residue using Hartree–Fock (Fock, 1930a, 1930b; Hartree, 1928a, 1928b) densities obtained with the 6‐31G* basis set (Ditchfield et al., 1971; Francl et al., 1982; Hariharan & Pople, 1973; Hehre et al., 1972), according to the standard AMBER recipe. The ESP values were computed on the default CHELPG (Breneman & Wiberg, 1990) grid available in NWChem while constraining the charges of all atoms in the acetyl group (ACE) and the amine group (NME) to the values reported in the respective force fields. For the FF99SB force field, the amide group charges were also constrained to reproduce the force field definition. Note that NWChem can only perform RESP fits for single conformations. Consequently, *PTM‐Psi* produces a Python script to obtain the multiple conformations fit using the data output by NWChem.

Step (3): Bond and angle force constants in the force fields for the non‐standard amino acids were generated with a modified Seminario method (Allen et al., 2018; Seminario, 1996) using the Hessian matrices of both α‐helix and β‐strand conformers computed at the same level of theory as the geometry optimizations described above. The default behavior was to obtain all the force constants in the ACE‐SNC‐NME dipeptide under the assumption that a chemical bond exists between two atoms separated by a distance smaller than 120% of the sum of the respective covalent radii (Cordero et al., 2008). The force constants computed by *PTM‐Psi* were compatible with the GROMACS software and given in kJ mol^−1^ nm^−2^ and kJ mol^−1^ rad^−2^ for bonds and angles, respectively. It is important to note that our implementation of the modified Seminario method used singular value decomposition of the non‐symmetric submatrices in order to avoid dealing with complex algebra. As a result, our method generally obtained stiffer bond constants, a direct consequence of Weyl's inequality theorem (Horn & Charles, 1991; Weyl, 1912).

Step (4): Torsion potentials for non‐standard amino acids were obtained by fitting KS‐DFT dihedral scans obtained with the r^2^SCAN‐D3(BJ) DFA and the def2‐SVP double‐ζ basis set. To avoid hysteresis during the QM dihedral scans, we relied on the TorsionDrive Python package (Qiu et al., 2020), which generates optimized structures on a grid of torsion constraints by means of a recursive wavefront propagation algorithm. Unlike other parameterization strategies where the torsion scans use only a small fragment of the dipeptide (Croitoru et al., 2021; Horton et al., 2022), the dihedral scans in *PTM‐Psi* are performed using full dipeptides in both α‐helix and β‐strand conformations. Our locally modified version of TorsionDrive is able to use NWChem as QM backend with several dihedral angles fixed. In our case, we fix *φ*, ψ, and the angle being scanned.

Step (5): Once the torsion scan information was available, *PTM‐Psi* uses the ForceBalance package (L. P. Wang et al., 2013, 2014) to fit the phases and force constants of the remainder dihedral potentials by directly comparing the potential energy surfaces from the MM and QM. The fit was performed while restraining the same three dihedrals with a force constant of at least 14,000 kJ mol^−1^ rad^−2^ (roughly equivalent to 1 kcal mol degree^−1^). The position of the rest of the atoms was weakly restrained with a force constant of 418 kJ mol^−1^ nm^−2^. These restraints allow some degree of relaxation in all orthogonal degrees of freedom while keeping the structure as close as possible to the QM scan. Our locally modified version of ForceBalance has been extended with a TorsionProfile_GMX target and uses the multiprocessing Python package to spawn as many child processes as there are physical cores available on a single computational node.

Step (6): The new amino acid was manually added to the aminoacids.itp file of the respective force field using the charges computed in Step (2). New atom types with their respective bonded parameters were manually added to the ffbonded.itp file whenever the data generated in Steps (3)–(5) presented substantial deviations from an equivalent atom already present in the force field.

### 5.4 MD simulations

#### 5.4.1 Generation of protein structure and topology

The GAP2 (PDB ID: 2D2I) (Kitatani et al., 2006) was PTMed using the modify command of the *PTM‐Psi* package. The pdb2gmx command of the GROMACS simulation package, version 2018.6, was used to generate the topology file with the modified AMBER FF99SB force field of Section 5.2.

#### 5.4.2 Setting up a simulation box

We used the TIP3P water model (Jorgensen et al., 1983) with Joung and Cheatham ion parameters (Joung & Cheatham, 2008) for the water molecules and ions for the system neutralization. The GAP2 was first solvated in a dodecahedron TIP3P water box with a buffer length of 1 nm, which is enough length to avoid self‐contacts between protein atoms across periodic boundary conditions.

#### 5.4.3 Adding ions to account for the screening effect

Accurately depicting electrostatic interactions in MD requires the correct number of ions in the simulation box to capture screening effects. We employed the *Screening Layer Tally by Container Average Potential* SLTCAP (Schmit et al., 2018) method developed by the Schmit group to mimic dilute conditions by considering only the volume of the water molecules, thereby excluding the protein volume. *PTM‐Psi* relies on residue‐specific volumetric data generated by means of Laguerre tessellation (Esque et al., 2010) in order to compute the protein volume.

#### 5.4.4 Equilibrations

The equilibrations started from solvent minimization while keeping the protein atoms restrained for 1000 1 kJ mol·nm^−2^. Subsequently, additional multistep minimizations were performed where harmonic restraints applied for the protein atoms were gradually reduced from 500, 200, 100, 10, 5, and 1 kJ mol·nm^−2^. Then, the system was equilibrated at 300 K for 500 ps in the constant‐temperature, constant‐volume (NVT) ensemble using the Berendsen velocity rescaling method followed by 500 ns equilibration at 1 atm and 300 K under a constant‐temperature, constant‐pressure (NPT) ensemble using the Berendsen pressure coupling method (Berendsen et al., 1984). The last minimization was carried out, and all the restraints applied on the protein atoms were removed during this minimization. Then, the system was re‐equilibrated without any restraints using the same procedures using NVT and NPT ensembles. After these equilibration steps, the systems were simulated for 100 ns at 300 K and 1 atm using the Parrinello–Rahman pressure coupling method (Parrinello & Rahman, 1981). Long‐range electrostatic interactions were evaluated using the particle mesh Ewald method (Darden et al., 1993). All hydrogen atoms were constrained using the Linear Constraints Solver (Hess et al., 1997). All simulations adopted a timestep of 2 fs. An identical procedure for the MD simulation has been successfully used for the MD simulations of other proteins with various mutations (H. Kim et al., 2022).

### 5.5 Free energy perturbation using thermodynamic integration

**FIGURE 14:**
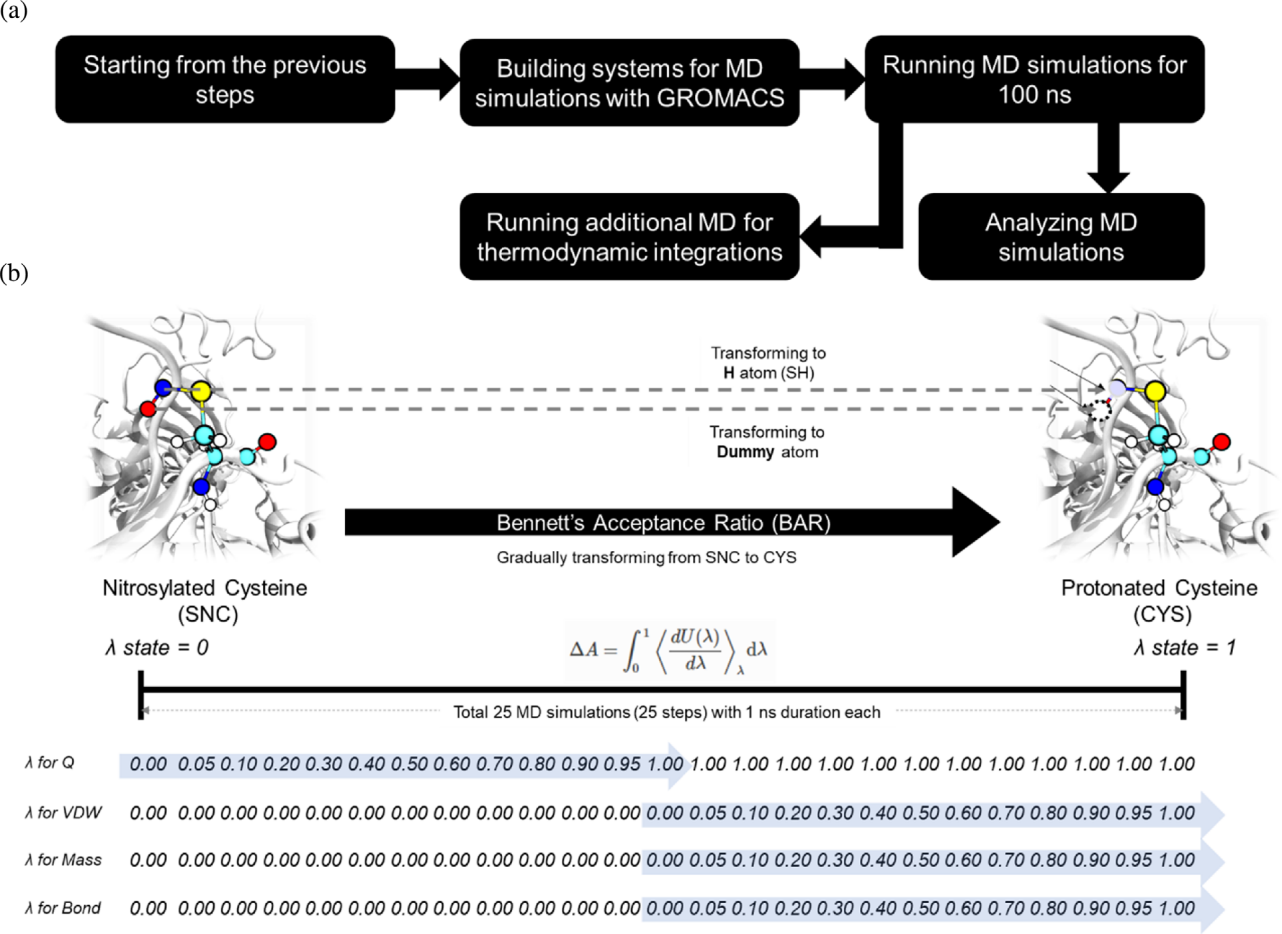
(a) The illustration of step (e) of *PTM‐Psi* from Figure 1 for the molecular dynamics (MD) simulations and analyses by free energy perturbation. (b) The subsequent procedure of MD for thermodynamic integration for free energy perturbation. A detailed description of the procedures is provided in the Section 5. *PTM‐Psi*, *p* ost‐ *t* ranslational *m* odification on *p* rotein *s* tructures and their *i* mpacts.

All MD simulations adopted a timestep of 2 fs. An identical procedure for the MD simulation has been successfully used for the MD simulations of other proteins with various mutations (H. Kim et al., 2022). Thermodynamic integration (TI) was performed to calculate the free energy differences before and after the PTM on selected cysteines. Specifically, we measured the free energy cost of transferring from a protonated state to its nitrosylated on the cysteine residue(s) of our interest. As for the TI, non‐bonded and bonded interactions were changed in a stepwise manner where first electrostatic followed by van der Waals interactions with the masses and bonded terms were decoupled by increasing *λ* from 0.00 (nitrosylated cysteine) to 1.00 (regular cysteine) in 13 steps (*λ* = 0.00, 0.05, 0.10, 0.20, …, 0.90, 0.95, 1.00), respectively. Thus, a total of 25 simulations of 1 ns duration each were employed for the calculation. Free energies were then obtained using Bennett's acceptance ratio method (Bennett, 1976) as implemented in the g_bar module of the GROMACS simulation package. Details about the workflows of the MD simulations and TI calculations are shown in Figure 14.

### 5.6 Molecular docking calculations

Autodock Vina (Eberhardt et al., 2021; Trott & Olson, 2010) and Autodock‐GPU (Santos‐Martins et al., 2021) software packages were used to perform the docking calculations. The NADP^+^ and GA3P structures were obtained from the respective SMILES strings in order to avoid any bias towards the crystal structure. The receptor PDBQT file was also obtained with the *PTM‐Psi* package using Meeko as the backend without further modifications. The search boxes were defined based on the bound NADP^+^ crystal structure with lengths of at least (19 Å × 27 Å × 16 Å) in order to accommodate both ligands. The Autodock Vina exhaustiveness parameter was set to 64. Atomic maps for the Autodock‐GPU calculations were generated using the *autogrid* program contained in the Autodock Suite. All other parameters were set at their default values.

### 5.7 Analysis of the simulation data

#### 5.7.1 Structural analysis

The CPPTRAJ module in the AmberTools18 package (Roe & Cheatham, 2013, 2018) and the analysis tools in the GROMACS 2018 package (Abraham et al., 2019) were mainly used for all post‐simulation analyses, such as close contact analyses, root mean square deviations, RMSFs, cluster analyses, distance analyses, and so on. Discovery Studio Visualizer (BIOVIA, 2017) and Visual Molecular Dynamics (Humphrey et al., 1996) were used for visually inspecting simulation trajectories and rendering the representative snapshots presented in this study. Protter was used for visualization of the protein sequence (Omasits et al., 2014).

#### 5.7.2 Non‐bonded close contact analysis

We performed the non‐bonded close contact analyses of the selected CYSs and adjacent amino acids and only counted them if certain amino acids satisfy the following three criteria (Figure 10a): (1) any atoms in the certain amino acids must be within 0.35 nm of given CYSs, and (2) retain these contacts at least 70% of the entire MD simulation, but (3) exclude any contacts with amino acids that make peptide bonds with CYSs (e.g., two amino acids right next to CYSs).

## Supporting information


**Figure S1.** Pie charts represent the contents of secondary structures.
