# Supplementary material for: PTM‐Psi : A python package to facilitate the computational investigation of p ost‐ t ranslational m odification on p rotein s tructures and their i mpacts on dynamics and functions
Source: Protein Sci. 2023 Dec 1;32(12):e4822. doi: 10.1002/pro.4822 (PMC10659954; doi:10.1002/pro.4822)
Supplement: Supplementary file 1 — Figure S1. Pie charts represent the contents of secondary structures. [file PRO-32-e4822-s001.docx]

PTM-Psi: A Python Package to Facilitate the Computational Investigation of Post-Translational Modification on Protein Structures and Their Impacts on Dynamics and Functions

Daniel Mejia-Rodriguez^^1^, Hoshin Kim^^1^, Natalie Sadler^2^, Xiaolu Li^2^, Pavlo Bohutskyi^2,3^, Marat Valiev^1^, Wei-Jun Qian^2^, Margaret S. Cheung^*1,4,5^

1. Physical Sciences Division, Physical and Computational Sciences Directorate, Pacific Northwest National Laboratory, Richland, WA
2. Biological Sciences Division, Earth and Biological Sciences Directorate, Pacific Northwest National Laboratory, Richland, WA
3. Biological Systems Engineering, Washington State University, Richland, WA
4. Environmental Molecular Sciences Laboratory, Richland, WA
5. University of Washington, Seattle, WA

Supplementary Figures


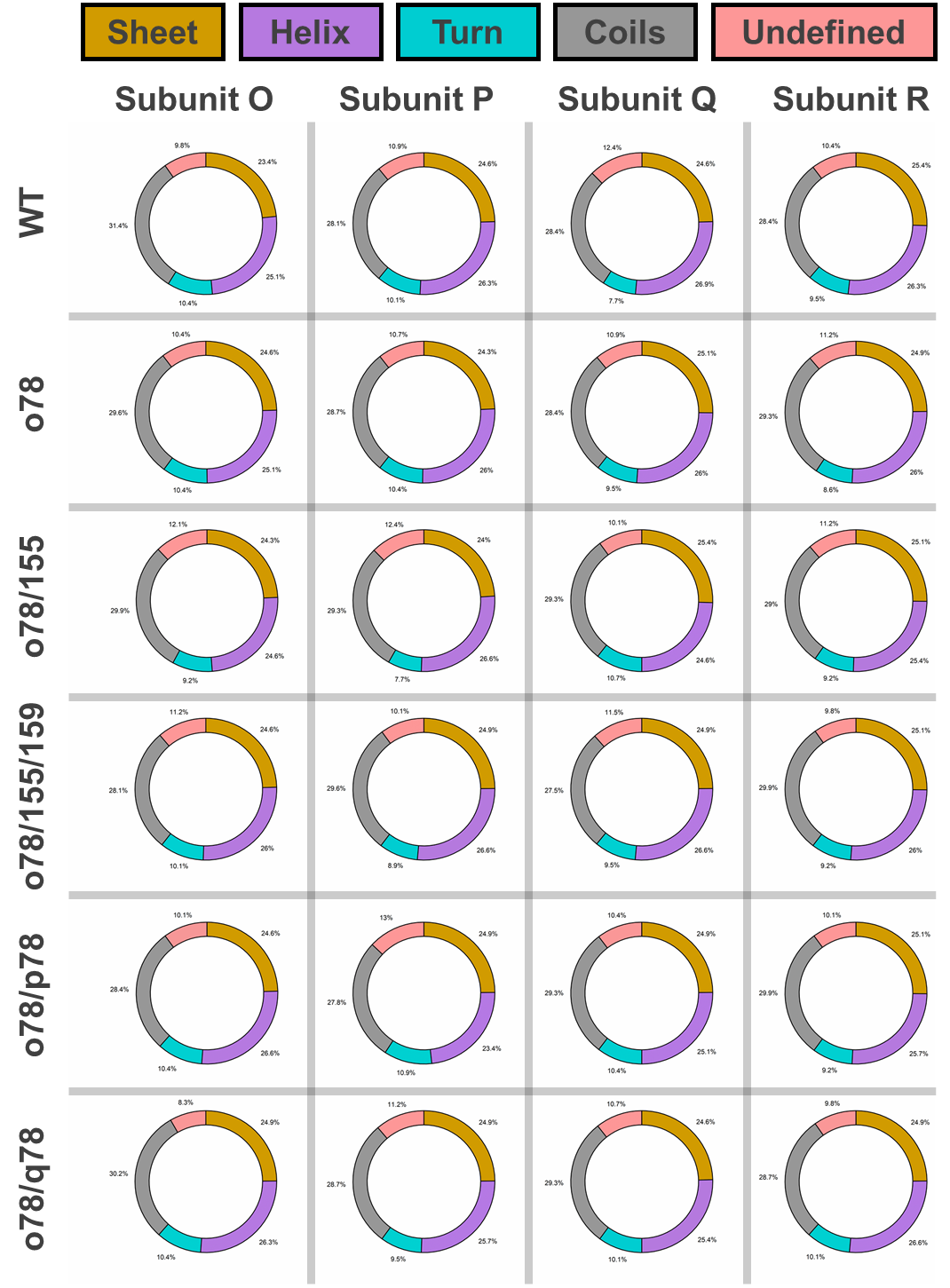


**Figure S1.** Pie charts represent contents of secondary structures in each subunit. If amino acids maintain certain secondary structural features greater than 70% of the entire simulations, they are counted. If amino acid has no certain features greater than 70%, they are considered as ‘undefined’. In the pie charts, brown, purple, cyan, dark grey, and pink color represent a sheet (both parallel and anti-parallel), a helix (both alpha, 3-10, and pi), a turn, a random coil, and an undefined one, respectively.
